# Identification of QTL for resistance to root rot in sweetpotato (*Ipomoea batatas* (L.) Lam) with SSR linkage maps

**DOI:** 10.1186/s12864-020-06775-9

**Published:** 2020-05-15

**Authors:** Zhimin Ma, Wenchuan Gao, Lanfu Liu, Minghui Liu, Ning Zhao, Meikun Han, Zhao Wang, Weijing Jiao, Zhiyuan Gao, Yaya Hu, Qingchang Liu

**Affiliations:** 1 Key Laboratory of Sweetpotato Biology and Biotechnology, Ministry of Agriculture and Rural Affairs/College of Agronomy and Biotechnology, China Agricultural University, Beijing, 100193 China; 2 Institute of Cereal and Oil Crops, Hebei Academy of Agriculture and Forestry Sciences/The Key Laboratory of Crop Genetics and Breeding of Hebei, Shijiazhuang, 050035 Hebei China; 3 Baoji Institute of Agriculture Science, Qishan, 722499 Shaanxi China

**Keywords:** Sweetpotato, Root rot, SSR marker, Linkage map construction, QTL analysis

## Abstract

**Background:**

Sweetpotato root rot is a devastating disease caused by *Fusarium solani* that seriously endangers the yield of sweetpotato in China. Although there is currently no effective method to control the disease, breeding of resistant varieties is the most effective and economic option. Moreover, quantitative trait locus (QTL) associated with resistance to root rot have not yet been reported, and the biological mechanisms of resistance remain unclear in sweetpotato. Thus, increasing our knowledge about the mechanism of disease resistance and identifying resistance loci will assist in the development of disease resistance breeding.

**Results:**

In this study, we constructed genetic linkage maps of sweetpotato using a mapping population consisting of 300 individuals derived from a cross between Jizishu 1 and Longshu 9 by simple sequence repeat (SSR) markers, and mapped seven QTLs for resistance to root rot. In total, 484 and 573 polymorphic SSR markers were grouped into 90 linkage groups for Jizishu 1 and Longshu 9, respectively. The total map distance for Jizishu 1 was 3974.24 cM, with an average marker distance of 8.23 cM. The total map distance for Longshu 9 was 5163.35 cM, with an average marker distance of 9.01 cM. Five QTLs (*qRRM_1*, *qRRM_2*, *qRRM_3, qRRM_4*, and *qRRM_5*) were located in five linkage groups of Jizishu 1 map explaining 52.6–57.0% of the variation. Two QTLs (*qRRF_1* and *qRRF_2*) were mapped on two linkage groups of Longshu 9 explaining 57.6 and 53.6% of the variation, respectively. Furthermore, 71.4% of the QTLs positively affected the variation. Three of the seven QTLs, *qRRM_3*, *qRRF_1*, and *qRRF_2*, were colocalized with markers IES43-5mt, IES68-6 fs**, and IES108-1 fs, respectively.

**Conclusions:**

To our knowledge, this is the first report on the construction of a genetic linkage map for purple sweetpotato (Jizishu 1) and the identification of QTLs associated with resistance to root rot in sweetpotato using SSR markers. These QTLs will have practical significance for the fine mapping of root rot resistance genes and play an important role in sweetpotato marker-assisted breeding.

## Background

Sweetpotato (*Ipomoea batatas* (L.) Lam.) is the seventh most important food crop in the world and also serves as raw materials in food and feed industries, and energy crops [1]. Sweetpotato root rot, caused by *Fusarium solani* [2], is one of the most widespread diseases in North China and directly affects sweetpotato production, resulting in yield losses and quality deterioration. In fact, this disease can lead to yield losses of 10–20%, and even 100% in severely infected fields [3]. There are currently no effective methodologies to control sweetpotato root rot. The breeding of resistant varieties is the most effective and economic way to control the disease. Conventional breeding for root rot resistance in sweetpotato is complicated, with a long cycle length, and generally improves only single traits. Combining molecular techniques with conventional breeding methods is an effective way to overcome the limitations of seasonal and environmental effects, species isolation, and linkage drag inherent to conventional breeding. However, root rot resistance loci have not been mapped in sweetpotato to date.

The construction of a genetic linkage map is imperative for the identification of quantitative trait locus (QTL), gene cloning, comparative genomic research, and marker-assisted selection breeding. However, sweetpotato, as a highly heterozygous, generally self-incompatible, and outcrossing hexaploid species with a large number of small chromosomes (2n = 6x = 90), poses numerous challenges for genetic analysis and breeding [4]. As a result, the progress of molecular biology research on sweetpotato lags far behind that made in other major crops.

Several genetic linkage maps for sweetpotato have been constructed using various molecular markers, including amplified fragment length polymorphism (AFLP), random amplified polymorphic DNA (RAPD), sequence-related amplified polymorphism (SRAP), simple sequence repeat (SSR), inter SSR, expressed sequence tag-SSR, retrotransposon insertion polymorphisms and single-nucleotide polymorphism (SNP) [5–17]. Ukoskit and Thompson constructed the first low-density linkage maps based on 196 RAPD markers from 76 progenies of the cross Vardaman × Regal [15]. Cervantes-Flores et al. developed genetic linkage maps of sweetpotato using AFLP markers, conducted the first QTL analysis for root knot nematode resistance, and identified 13 QTLs for dry matter content, 12 QTLs for starch content, eight QTLs for β-carotene content [5, 18, 19]. Zhao et al. developed the first map that included 90 complete sweetpotato linkage groups based on AFLP and SSR markers, and mapped 27 QTLs for storage root dry matter content [17]. Using this map, Yu et al. and Li et al. identified QTLs and colocalizing markers for starch content and storage root yield [20, 21].

With the development of high-throughput technology, next-generation sequencing (NGS) has been used to analyse genetic linkages in numerous crop species. For instance, using NGS, Shirasawa et al. established the first high-density genetic map for sweetpotato using SNPs identified by double-digest restriction site-associated DNA sequencing to construct a map for Xushu 18 using an S_1_ mapping population comprising 142 individuals, which had 28,087 double-simplex SNPs mapped onto 96 linkage groups, and covered a total distance of 33,020.4 cM [13]. Furthermore, Mollinari et al. constructed an ultradense multilocus integrated genetic map and characterized the inheritance system in a sweetpotato full-sib family using a newly developed software, MAPpoly [10].

In the present study, we used a mapping population of 300 F_1_ individuals derived from a cross between Jizishu 1 and Longshu 9 to construct linkage maps using SSR markers and to conduct QTL analysis for resistance to root rot in sweetpotato. The results of this study are expected to provide useful information for developing resistance to root rot based on major QTLs.

## Results

### Genetic linkage map construction

In total, 155 primer pairs (Additional file 3: Table S1) were polymorphic in the parents and ten progenies and were selected to analyse the F_1_ population. Finally, 839 high-quality polymorphic markers were obtained, with an average of five markers per primer pair. In total, 506 polymorphic SSR markers were obtained for mapping Jizishu 1, including 217 simplex, 47 duplex, 8 triplex, and 234 double-simplex markers, and 567 polymorphic SSR markers were obtained for mapping Longshu 9, including 237 simplex, 76 duplex, 20 triplex, and 234 double-simplex markers. The percentage of simplex markers was 79.8% (217/(217 + 47 + 8)) and 71.2% (237/(237 + 76 + 20)) in Jizishu 1 and Longshu 9, respectively, which was in accordance with the theoretical values for an autohexaploid (75% simplex and 25% non-simplex) according to Chi-square analysis results, and could be used to construct a genetic map of the hexaploid sweetpotato [5, 8, 17].

The single-dose markers were used to construct a framework map of each parent at a LOD score of 5.0 using JoinMap 4.0 software [22]. Subsequently, duplex and triplex markers were inserted into the framework maps to obtain the final genetic linkage maps. Molecular markers were grouped into 90 linkage groups for each parental map. There were 54 major and 36 minor groups of three or two markers for Jizishu 1, and 68 major and 22 minor groups for Longshu 9.

The linkage map of Jizishu 1 was composed of 484 polymorphic markers, of which 186, 137, 30, and 131 were simplex, duplex, triplex and double-simplex markers, respectively. The largest linkage group contained 17 markers, while the smallest group contained 2 markers. The total map distance was 3974.24 cM, with an average marker distance of 8.23 cM. The longest linkage group was 143.52 cM, the shortest was 0.34 cM, and the average linkage group length was 44.16 cM (Table 1). Moreover, the linkage map of Longshu 9 was composed of 573 polymorphic markers, of which 185, 217, 40, and 131 were simplex, duplex, triplex and double-simplex markers, respectively. The largest and smallest linkage groups contained 17 and 2 markers, respectively. The total map distance was 5163.35 cM, with an average marker distance of 9.01 cM. The longest linkage group was 151.60 cM, the shortest was 4.07 cM, and the average linkage group length was 57.37 cM (Table 2). There were 239 (49.38%) and 250 distorted markers (43.63%) in Jizishu 1 and Longshu 9, respectively.

**Table 1:** Distribution of SSR markers in Jizishu 1 genetic linkage maps

Linkage group	Type of markers				No. of markers	No. of segregation distortion	Map length (cM)	Average distance (cM)
	Simplex	Duplex	Triplex	Double-simplex				
JZ1(01.01)	9	6	1	1	17	6	143.52	8.44
JZ1(01.02)	4	6	1	0	11	3	40.40	3.67
JZ1(01.03)	5	1	1	3	10	6	94.04	9.40
JZ1(01.04)	0	7	1	2	10	3	36.46	3.65
JZ1(01.05)	5	1	0	0	6	6	56.33	9.39
JZ1(01.06)	0	6	1	1	8	2	70.69	8.84
JZ1(02.07)	0	5	4	1	10	5	83.04	8.30
JZ1(02.08)	2	2	0	4	8	6	94.53	11.82
JZ1(02.09)	0	9	4	2	15	8	127.36	8.49
JZ1(02.10)	5	1	0	1	7	4	82.11	11.73
JZ1(02.11)	6	1	1	1	9	6	100.24	11.14
JZ1(02.12)	0	1	0	3	4	3	16.65	4.16
JZ1(03.13)	0	5	4	4	13	6	99.14	7.63
JZ1(03.14)	0	7	4	3	14	7	89.89	6.42
JZ1(03.15)	3	2	0	2	7	3	77.80	11.11
JZ1(03.16)	0	2	0	2	4	3	30.76	7.69
JZ1(03.17)	0	6	4	1	11	5	96.60	8.78
JZ1(03.18)	3	1	0	0	4	4	43.73	10.93
JZ1(04.19)	1	13	0	3	17	6	114.42	6.73
JZ1(04.20)	0	1	0	3	4	3	26.34	6.59
JZ1(04.21)	3	1	0	2	6	1	71.29	11.88
JZ1(04.22)	4	3	1	0	8	4	81.43	10.18
JZ1(04.23)	2	2	0	1	5	0	79.21	15.84
JZ1(04.24)	0	3	0	2	5	1	62.64	12.53
JZ1(05.26)	0	1	0	3	4	2	53.60	13.40
JZ1(05.27)	3	2	0	1	6	5	54.03	9.01
JZ1(05.28)	4	1	0	0	5	4	64.42	12.88
JZ1(05.29)	0	3	0	5	8	2	69.87	8.73
JZ1(05.30)	3	1	0	1	5	2	58.26	11.65
JZ1(06.31)	1	6	0	5	12	1	97.33	8.11
JZ1(06.32)	4	6	0	0	10	4	71.89	7.19
JZ1(06.33)	0	6	0	3	9	3	34.17	3.80
JZ1(06.34)	0	6	0	1	7	0	58.09	8.30
JZ1(07.35)	6	2	1	0	9	1	91.66	10.18
JZ1(07.36)	0	1	0	3	4	3	34.73	8.68
JZ1(07.37)	0	1	0	2	3	2	10.84	3.61
JZ1(08.38)	2	1	0	3	6	6	57.42	9.57
JZ1(08.39)	3	1	0	1	5	2	47.16	9.43
JZ1(00.40)	0	0	0	4	4	2	47.20	11.80
JZ1(00.41)	1	0	0	1	2	1	13.26	6.63
JZ1(00.42)	4	0	0	0	4	1	19.78	4.95
JZ1(00.43)	2	0	0	0	2	0	0.34	0.17
JZ1(00.44)	0	0	0	5	5	5	58.76	11.75
JZ1(00.45)	1	0	0	1	2	1	19.76	9.88
JZ1(00.46)	1	0	0	1	2	2	4.57	2.29
JZ1(00.47)	3	0	0	0	3	1	26.56	8.85
JZ1(00.48)	3	0	0	0	3	0	50.48	16.83
JZ1(00.49)	0	0	0	2	2	2	13.18	6.59
JZ1(00.50)	1	0	0	2	3	1	15.86	5.29
JZ1(00.51)	2	0	0	0	2	0	19.69	9.85
JZ1(00.52)	10	1	0	0	11	6	115.20	10.47
JZ1(00.53)	0	0	0	6	6	6	20.52	3.42
JZ1(00.54)	5	0	0	0	5	4	80.27	16.05
JZ1(00.55)	2	1	0	1	4	4	61.71	15.43
JZ1(00.56)	1	0	0	1	2	2	24.59	12.30
JZ1(00.57)	0	0	0	3	3	3	6.23	3.12
JZ1(00.58)	6	1	0	2	9	3	59.84	6.65
JZ1(00.59)	2	0	0	0	2	2	17.78	8.89
JZ1(00.60)	1	0	0	2	3	2	10.04	3.35
JZ1(00.61)	4	0	0	0	4	0	10.83	2.71
JZ1(00.62)	2	1	0	0	3	2	52.71	17.57
JZ1(00.63)	0	1	0	3	4	3	27.56	6.89
JZ1(00.64)	5	0	0	0	5	1	14.75	2.95
JZ1(00.65)	5	0	0	0	5	2	14.01	2.80
JZ1(00.66)	4	0	0	0	4	1	90.45	22.61
JZ1(00.67)	4	0	0	0	4	4	53.42	13.36
JZ1(00.68)	3	0	0	0	3	3	45.10	15.03
JZ1(00.69)	0	0	0	2	2	2	41.70	20.85
JZ1(00.70)	1	0	0	3	4	1	13.74	3.44
JZ1(00.71)	3	0	0	1	4	2	13.47	3.37
JZ1(00.72)	1	0	0	2	3	2	38.51	12.84
JZ1(00.73)	0	0	0	3	3	3	10.61	3.54
JZ1(00.74)	3	0	0	0	3	2	4.09	1.36
JZ1(00.75)	2	0	0	1	3	1	13.05	4.35
JZ1(00.76)	2	0	0	1	3	1	4.01	1.34
JZ1(00.77)	3	0	0	0	3	1	13.93	4.64
JZ1(00.78)	3	0	0	0	3	1	16.21	5.40
JZ1(00.79)	3	0	0	0	3	2	7.29	2.43
JZ1(00.80)	2	0	0	1	3	0	33.87	11.29
JZ1(00.81)	3	0	0	0	3	1	21.95	7.32
JZ1(00.82)	0	0	0	2	2	2	0.48	0.24
JZ1(00.83)	0	0	0	2	2	2	9.38	4.69
JZ1(00.84)	2	0	0	0	2	2	0.69	0.35
JZ1(00.85)	2	0	0	0	2	2	13.15	6.58
JZ1(00.86)	1	0	0	1	2	0	5.82	2.91
JZ1(00.87)	2	0	0	0	2	2	1.71	0.86
JZ1(00.88)	1	0	0	1	2	2	4.44	2.22
JZ1(00.89)	2	0	0	0	2	0	4.22	2.11
JZ1(00.90)	0	0	0	2	2	2	0.47	0.23
Total	186	137	30	131	484	239	3974.24	8.23

**Table 2:** Distribution of SSR markers in Longshu 9 genetic linkage maps

Linkage group	Type of markers				No. of markers	No. of segregation distortion	Map length (cM)	Average distance (cM)
	Simplex	Duplex	Triplex	Double-simplex				
L9(01.01)	2	8	0	0	10	2	85.36	8.54
L9(01.02)	6	4	0	0	10	5	95.48	9.55
L9(01.03)	2	13	0	0	15	4	126.65	8.44
L9(01.04)	0	10	1	6	17	7	105.80	6.22
L9(01.05)	0	1	0	5	6	2	66.71	11.12
L9(01.06)	0	11	1	1	13	2	106.30	8.18
L9(02.07)	0	9	2	5	16	3	146.46	9.15
L9(02.08)	0	1	0	4	5	2	51.04	10.21
L9(02.09)	0	7	4	1	12	0	73.46	6.12
L9(02.10)	3	5	6	2	16	3	60.15	3.76
L9(02.11)	0	2	0	3	5	3	45.96	9.19
L9(02.12)	2	8	7	0	17	2	85.78	5.05
L9(03.13)	0	7	2	7	16	8	94.87	5.93
L9(03.14)	3	2	2	3	10	3	102.42	10.24
L9(03.15)	2	1	0	1	4	3	36.56	9.14
L9(03.16)	10	3	0	1	14	10	102.07	7.29
L9(03.17)	2	1	0	0	3	2	30.29	10.10
L9(03.18)	0	7	0	1	8	1	84.66	10.58
L9(04.19)	0	1	0	3	4	3	32.79	8.20
L9(04.20)	12	3	1	0	16	11	151.60	9.48
L9(04.21)	1	1	1	0	3	1	36.35	12.12
L9(04.22)	5	1	1	1	8	5	134.20	16.78
L9(04.23)	2	1	0	2	5	3	48.77	9.75
L9(04.24)	0	2	0	2	4	4	12.99	3.25
L9(05.25)	9	5	0	1	15	7	150.50	10.03
L9(05.26)	0	4	0	0	4	0	11.35	2.84
L9(05.27)	0	4	0	1	5	1	23.37	4.67
L9(05.28)	4	4	0	2	10	4	97.44	9.74
L9(05.29)	0	5	0	3	8	3	49.81	6.23
L9(05.30)	0	1	0	2	3	2	6.84	2.28
L9(06.31)	0	6	0	1	7	1	61.26	8.75
L9(06.32)	2	4	0	0	6	3	64.99	10.83
L9(06.33)	3	1	1	0	5	4	56.77	11.35
L9(06.34)	2	8	0	2	12	5	100.20	8.35
L9(06.35)	0	1	0	3	4	0	51.52	12.88
L9(06.36)	1	3	0	1	5	3	64.77	12.95
L9(07.37)	2	1	0	1	4	3	46.29	11.57
L9(07.38)	0	6	2	0	8	2	52.75	6.59
L9(07.39)	0	5	0	0	5	2	63.37	12.67
L9(07.40)	3	5	0	0	8	1	76.56	9.57
L9(07.41)	2	4	0	0	6	4	89.69	14.95
L9(07.42)	0	6	1	2	9	3	54.87	6.10
L9(08.43)	0	8	1	2	11	4	85.64	7.79
L9(08.44)	0	3	0	1	4	3	77.82	19.46
L9(08.45)	1	0	1	5	7	5	59.32	8.47
L9(08.46)	0	1	0	3	4	3	46.02	11.51
L9(08.47)	0	5	1	3	9	4	30.52	3.39
L9(08.48)	0	1	0	3	4	3	43.63	10.91
L9(09.49)	6	1	0	0	7	1	74.91	10.70
L9(09.50)	4	1	0	0	5	2	74.49	14.90
L9(09.51)	1	2	1	0	4	2	53.73	13.43
L9(09.52)	0	2	2	3	7	3	60.82	8.69
L9(09.53)	0	3	0	2	5	2	43.12	8.62
L9(09.54)	0	3	1	2	6	3	17.31	2.89
L9(00.55)	5	0	0	3	8	4	79.75	9.97
L9(00.56)	4	0	0	0	4	1	37.16	9.29
L9(00.57)	0	2	0	5	7	4	81.69	11.67
L9(00.58)	6	0	0	0	6	3	47.11	7.85
L9(00.59)	0	0	0	5	5	3	56.99	11.40
L9(00.60)	3	1	0	0	4	3	84.75	21.19
L9(00.61)	0	0	0	2	2	2	13.18	6.59
L9(00.62)	3	1	0	0	4	1	50.95	12.74
L9(00.63)	3	0	0	1	4	3	51.61	12.90
L9(00.64)	4	0	0	2	6	4	62.34	10.39
L9(00.65)	4	0	0	2	6	3	58.10	9.68
L9(00.66)	4	0	0	1	5	2	53.29	10.66
L9(00.67)	5	0	0	0	5	3	58.30	11.66
L9(00.68)	3	0	0	0	3	2	28.83	9.61
L9(00.69)	3	0	0	0	3	2	18.92	6.31
L9(00.70)	3	0	0	1	4	3	43.99	11.00
L9(00.71)	1	0	0	1	2	1	16.97	8.49
L9(00.72)	3	0	0	3	6	4	77.31	12.89
L9(00.73)	5	0	0	1	6	5	56.25	9.38
L9(00.74)	3	0	0	1	4	1	51.52	12.88
L9(00.75)	2	0	1	3	6	5	52.62	8.77
L9(00.76)	3	0	0	0	3	1	48.55	16.18
L9(00.77)	3	0	0	0	3	1	45.59	15.20
L9(00.78)	0	0	0	3	3	3	12.94	4.31
L9(00.79)	2	0	0	1	3	2	8.60	2.87
L9(00.80)	3	0	0	0	3	0	44.83	14.94
L9(00.81)	3	0	0	0	3	3	33.75	11.25
L9(00.82)	3	0	0	0	3	0	25.99	8.66
L9(00.83)	3	0	0	0	3	2	54.05	18.02
L9(00.84)	1	0	0	1	2	1	14.03	7.02
L9(00.85)	2	0	0	0	2	1	13.09	6.55
L9(00.86)	1	0	0	1	2	1	27.62	13.81
L9(00.87)	0	1	0	2	3	2	5.00	1.67
L9(00.88)	2	0	0	0	2	0	26.90	13.45
L9(00.89)	2	0	0	0	2	1	10.31	5.16
L9(00.90)	1	0	0	1	2	1	4.07	2.04
Total	185	217	40	131	573	250	5163.35	9.01

For Jizishu 1, 132 duplex and 30 triplex markers divided 39 homologous relationships into 8 homologous linkage groups. The remaining 51 linkage groups could not be classified into any homologous linkage group (Additional file 1: Fig. S1). For Longshu 9, 212 duplex and 39 triplex markers divided 54 homologous relationships into 9 homologous linkage groups. The remaining 36 linkage groups could not be classified into any homologous linkage group (Additional file 2: Fig. S2).

Double-simplex markers were used to detect the homology of the corresponding linkage groups in the two maps. Among them, 100 double-simplex markers revealed that 42 linkage groups in Jizishu 1 map had homologous linkage relationships with 40 linkage groups in Longshu 9 map (Additional file 4: Table S2). Homology between the two parental maps is an important criterion for consistency of the maps.

### QTL analysis

**Fig. 1:**
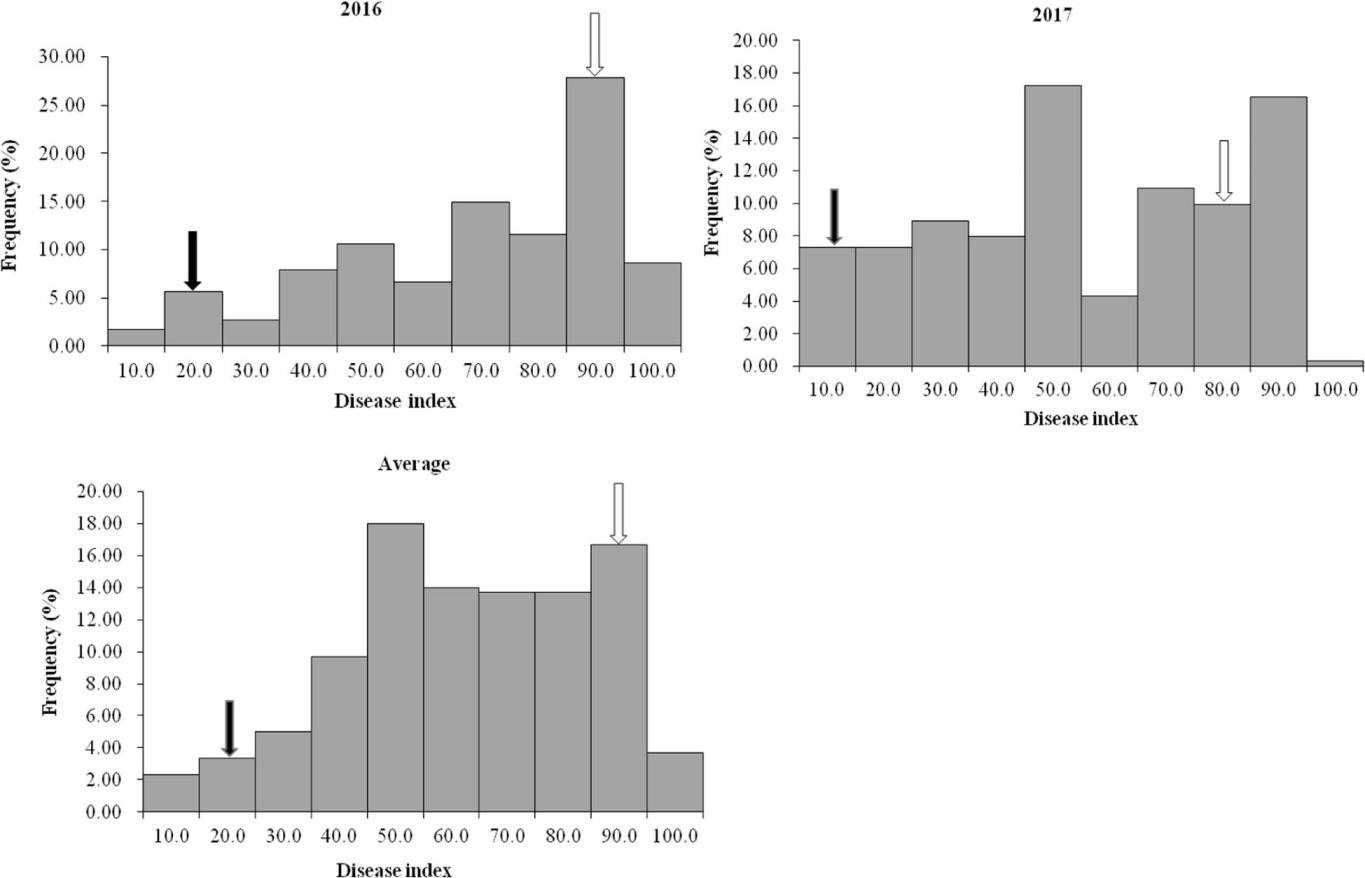
Frequency distribution of disease index for sweetpotato root rot in the mapping population. Black and white arrows indicate the disease index of Jizishu 1 and Longshu 9, respectively

The root rot disease index in the mapping population showed abnormal distributions in 2016 and 2017 (Fig. 1), with the average disease index of the mapping population ranging from 3.2 to 100, and a population mean of 58.4. The average disease index of Jizishu 1 was 14.4, indicating high resistance to root rot, and the average disease index of Longshu 9 was 84.5, indicating high susceptibility. Furthermore, ANOVA showed that the disease index differed significantly between the two years (Table 3). Therefore, the disease index for each year, and the average values were analysed separately for QTL mapping. In addition, transgressive segregation was observed, that is, certain progenies showed a higher disease index, while other exhibited a lower disease index compared to either parent.

**Table 3:** Anova of the disease index in the mapping population of Jizishu 1 × Longshu 9

Source	*df*	SS	MS (SS/*df*)	F value	*P* value
Year	1	1,781,466.431	1,781,466.431	2015.524	.000
Error	267	235,993.941	883.827		

*df* degrees of freedom

*SS* sum of squares

*MS* mean sum of squares

**Fig. 2:**
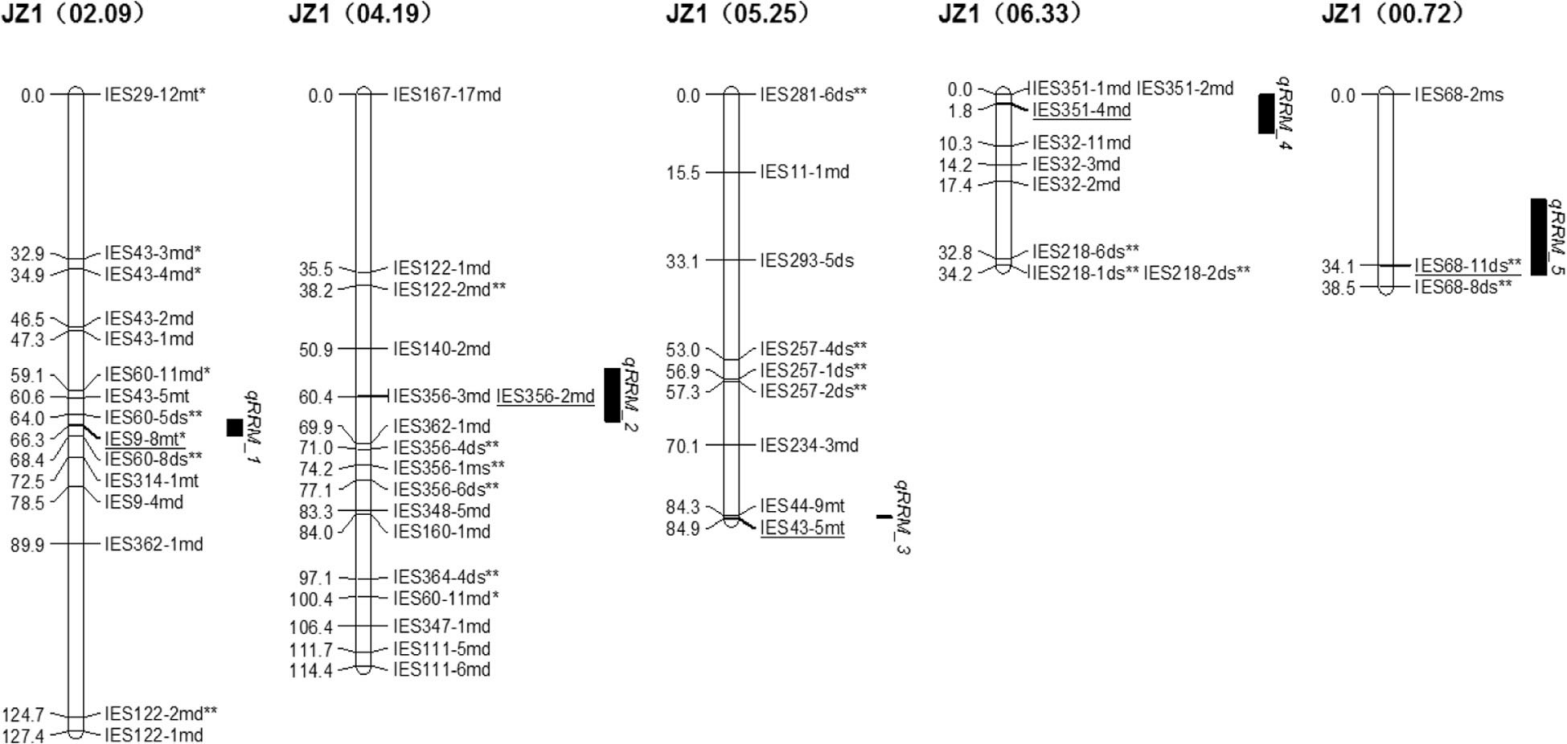
QTLs for resistance to root rot identified in the Jizishu 1 linkage groups. QTLs were shown as vertical bars on the right side of the respective linkage groups. The QTL corresponding markers were indicated by underlined text

**Fig. 3:**
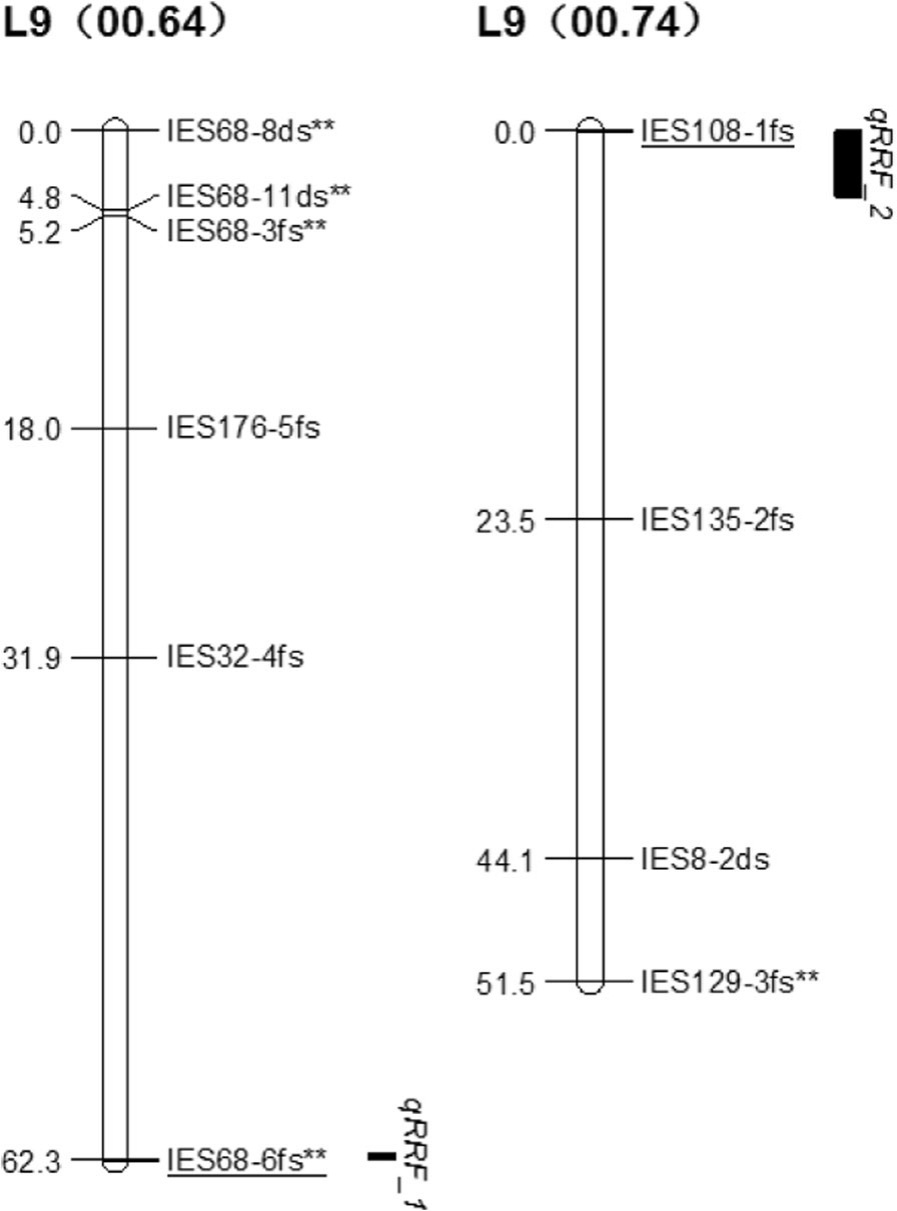
QTLs for resistance to root rot identified in the Longshu 9 linkage groups. QTLs were shown as vertical bars on the right side of the respective linkage groups. The QTL corresponding markers were indicated by underlined text

Seven stable QTLs were identified for resistance to root rot at the same genomic location in 2016, 2017, and in the average data (Table 4). Five QTLs for root rot resistance, *qRRM_1*, *qRRM_2*, *qRRM_3, qRRM_4*, and *qRRM_5* were located in five linkage groups of Jizishu 1, JZ1 (02.09), JZ1 (04.19), JZ1 (05.25), JZ1 (06.33), and JZ1 (00.72), respectively, and explained 52.6–57.0% of the variation in root rot resistance (Table 4 and Fig. 2). Among the five QTLs, only *qRRM_4* had a negative effect on resistance to root rot, explaining 57.0% of the variation, whereas the remaining four QTLs exhibited a positive effect on resistance. Two QTLs, *qRRF_1* and *qRRF_2*, were located in two linkage groups of Longshu 9, L9 (00.64) and L9 (00.74), respectively (Fig. 3). *qRRF_1* exerted a positive, while *qRRF_2* had a negative effect on root rot resistance, explaining 57.6 and 53.6% of the variation, respectively (Table 4). These results verify that Jizishu 1 is highly resistant, whereas Longshu 9 is highly susceptible to root rot.

**Table 4:** QTLs detected for resistance to root rot in the Jizishu 1 × Longshu 9 mapping population

QTL	Linkage group	Marker	Marker position (cM)^a^	QTL position (cM)^b^	Environment	LOD^c^	R^2^(%)^d^
*qRRM_1*^p^	JZ1(02.09)	IES9-8mt*	66.331	67.331	Y2016	5.04	65.0
				66.045	Y2017	10.15	67.1
				66.045	AVERAGE	3.83	55.9
*qRRM_2*^p^	JZ1(04.19)	IES356-2md	60.406	63.406	Y2016	3.59	60.7
				63.406	Y2017	8.22	63.0
				63.406	AVERAGE	5.55	53.4
*qRRM_3*^p^	JZ1(05.25)	IES43-5mt	84.907	84.907	Y2016	3.48	65.2
				84.907	Y2017	8.95	65.5
				84.907	AVERAGE	3.31	56.5
*qRRM_4*^n^	JZ1(06.33)	IES351-4md	1.844	4.844	Y2016	4.51	63.2
				2.844	Y2017	6.29	65.0
				2.844	AVERAGE	3.48	57.0
*qRRM_5*^p^	JZ1(00.72)	IES68-11ds**	34.102	31.000	Y2016	9.58	62.7
				34.102	Y2017	4.12	58.9
				34.102	AVERAGE	3.85	52.6
*qRRF_1*^p^	L9(00.64)	IES68-6 fs**	62.342	62.342	Y2016	5.04	65.0
				62.342	Y2017	7.00	65.9
				62.342	AVERAGE	3.00	57.6
*qRRF_2*^n^	L9(00.74)	IES108-1 fs	0.000	0.000	Y2016	5.78	65.5
				0.000	Y2017	6.95	63.9
				0.000	AVERAGE	3.77	53.6

^a^the closely linked or co-localized markers position

^b^LOD peak position

^c^the estimated LOD score at the QTL peak

^d^proportion of phenotypic variation explained by the QTL

^p^QTL with a positive effect on resistance to root rot

^n^QTL with a negative effect on resistance to root rot

*the distorted markers indicate significant differences at the 0.05 level

**the distorted markers indicate significant differences at the 0.01 level

At the location with the highest LOD scores, three of the seven QTLs (*qRRM_3*, *qRRF_1* and *qRRF_2*) were colocalized with the markers IES43-5mt, IES68-6 fs**, and IES108-1 fs. Moreover, *qRRM_1*, *qRRM_2*, *qRRM_4*, and *qRRM_5* were closely linked to IES9-8mt*, IES356-2md, IES351-4md, and IES68-11ds**, respectively. These QTLs and their colocalized markers could be used for marker-assisted selection of resistance to root rot in sweetpotato.

## Discussion

When generating a genetic population, the genetic characteristics and differences among the parents should be thoroughly considered. Within a certain range, a higher level of polymorphism can be detected when the parents are distantly related and have greater genetic differences, and hence, the constructed map will be more accurate and more saturated. Jizishu 1 is a cultivar with purple skin, purple flesh, high starch content, and high resistance to root rot. Alternatively, Longshu 9 is a fresh-eating cultivar with red skin, yellow flesh, low starch content, and high susceptibility to root rot. The genetic variation between these two cultivars is high, and the cross was suitable for constructing a mapping population. As the difference in disease resistance was significant, the QTLs for root rot resistance could be located.

Only four genetic linkage maps of sweetpotato based on SSR markers have been reported, two which were constructed by EST-SSR markers. Tang et al. constructed the first EST-SSR-based genetic linkage maps with a mapping population of 189 progenies. In total, 74 linkage groups for the female parent, constructed based on 215 loci, were placed on the genetic linkage map. The linkage map covered a total length of 3826.07 cM, with an average inter-marker distance of 17.80 cM. A genetic linkage map for the male parent was constructed using 250 loci distributed on 80 linkage groups. The linkage map covered 3955.0 cM, with an average inter-marker distance of 15.7 cM. Seventeen QTLs for starch content were identified [14]. Similarly, Kim et al. constructed a genetic linkage map based on 137 progenies derived from ‘Yeseumi’ and ‘Annobeny’ with 210 EST-SSR markers. The total length was 1508.1 cM, and the mean distance between markers was 7.2 cM. In addition, three QTLs for internode length, one for skin thickness, 15 for bare skin main colour, and two for bare skin secondary colour were identified in the genetic linkage maps [7]. Zhao et al. constructed a high-density linkage map based on AFLP and SSR markers using an F_1_ population comprising 202 individuals derived from a cross between Xushu 18 and Xu 781. The Xushu 18 map included 1936 AFLP and 141 SSR markers, while the Xu 781 map contained 1824 AFLP and 130 SSR markers, with the SSR markers accounting for only 6.7% of the total markers [17]. An additional high-density linkage map was constructed based on the retrotransposon insertion polymorphisms, SSR, and SNP markers by Sasai et al., however, only 161 and 176 SSR markers were identified in the female parent and male parent, respectively [12].

In our study, 484 and 573 SSR polymorphic markers were grouped into 90 linkage groups on each parental map, respectively. The total map distance of Jizishu 1 was 3974.24 cM, with an average marker distance of 8.23 cM, while that of Longshu 9 was 5163.35 cM, with an average marker distance of 9.01 cM. Compared with previously constructed maps, these contain more polymorphic SSR markers and linkage groups, as well as longer total map distances, and shorter average inter-marker distances. Moreover, this is the first study to construct a genetic linkage map for purple sweetpotato (Jizishu 1) based on SSR markers, which can be used to enrich the genetic maps of and improve the genetic research on different sweetpotato varieties.

Similar to findings reported by Cervantes-Flores et al. [5], Kriegner et al. [8], Li et al. [9], and Zhao et al. [17], herein, 239 distorted markers (49.38%) and 250 distorted markers (43.63%) were detected in genetic linkage maps of Jizishu 1 and Longshu 9, respectively. This suggests that preferential pairing occurs in sweetpotato. There are many reasons for distorted segregation. For instance, genetic factors such as gamete fertility, non-homologous recombination, gene conversion, and transposons can affect the distribution of marker sites in a population [23]. In addition, environmental factors and human intervention may cause distorted segregation. For example, the possibility of abnormal segregation appeared in population construction [24]. Sweetpotato is a highly heterozygous vegetative reproduction species, and its offspring are genetically unstable. An impure gene locus controlling a certain character may also cause distorted segregation in some lines [25].

Transgressive segregation was also observed for the root rot disease index in the mapping population. This may be due to the high heterozygosity of the parents, which results in the loss or accumulation of favourable alleles in offspring [19, 26, 27]. In sweetpotato breeding programs, this is commonly observed, particularly in the hybrid offspring of parental materials with significant genetic differences.

The distribution frequencies of the disease index in the F_1_ progeny were continuous, however, abnormal in this study. We mapped seven stable QTLs, which explained 52.6–57.0% of the phenotypic variation in root rot resistance. Accordingly, we speculated that the root rot resistance of sweetpotato may be controlled by several major QTLs. Similarly, Agarwal et al. mapped two major-effect QTLs for early leaf spot, which explained 47.42 and 47.38% of the phenotypic variation, and two QTLs for late leaf spot, explaining 47.63 and 34.03% of phenotypic variation in peanut [28]. Moreover, Kumar et al. revealed three significant QTLs for resistance to loose smut in tetraploid durum wheat, one of which explained up to 74% of phenotypic variation [29]. Using composite interval mapping and inclusive composite interval mapping, two major QTLs and one minor QTL were validated which had significant effects in reducing stripe rust severity and explained 59.0–74.1% of the phenotype variation in disease response [30]. However, due to the limited number of markers on the genetic map, it is necessary to increase the density of markers on the map to accurately locate the genes against root rot.

Of the seven QTLs, five were mapped on the Jizishu 1 map, 80.0% of which had a positive effect, and two were located on the Longshu 9 map, one of which had a positive effect. These results confirm that Jizishu 1 is substantially more resistant to root rot than Longshu 9. Furthermore, *qRRM_3*, *qRRF_1*, and *qRRF_2* were colocalized with the corresponding markers IES43-5mt, IES68-6 fs**, and IES108-1 fs. Due to the limited number of markers on the linkage map, the accuracy rate of the three markers in the segregating population was only 51, 51, and 47%, respectively. In the future, we will develop more markers to encrypt the linkage maps, mine more accurate colocalized markers, and construct a natural segregating population to test the accuracy rate. These QTLs identified in this study will have practical significance for gene cloning, and genome research of sweetpotato. Because of the lack of QTL mapping data for root rot resistance in sweetpotato in the literature, it is difficult to verify these QTLs. Therefore, the trait will be further studied and monitored in future studies to verify the loci identified in this research.

## Conclusions

In this study, the first genetic linkage maps of purple sweetpotato (Jizishu 1) were constructed by SSR markers. To our knowledge, this is also the first report on the identification of QTLs associated with resistance to root rot in sweetpotato. These results will have practical significance for the fine mapping of root rot resistance genes and marker-assisted selection breeding for sweetpotato.

## Methods

### Plant materials

The mapping population was derived from a cross between the female parent Jizishu 1, a cultivar with resistance to root rot and high starch content that is popular in north China, and the male parent Longshu 9, a cultivar that is susceptible to root rot, has high yield and low starch content, and is popular in China. Both parents and the F_1_ generation were collected from the Institute of Cereal and Oil Crops, Hebei Academy of Agriculture and Forestry Sciences (Shijiazhuang, China) and analysed for esterase isozymes, self-bred progenies were deleted. In total, 300 progenies were used for genetic linkage map construction and QTL analysis.

### DNA extraction

Genomic DNA was extracted from fresh leaves of Jizishu 1, Longshu 9, and the 300 F_1_ individuals using the cetyltrimethylammonium bromide method [31, 32]. DNA concentrations and quality were determined using an ultraviolet spectrophotometer (NanoDrop 2000, Thermo Fisher Scientific, USA) and 1.0% agarose gel electrophoresis respectively. The DNA was diluted to 50 ng/μL.

### Genotyping

PCR was carried out using 20 μL reaction mixtures containing 1 μL of DNA (50 ng/μL), 0.6 μL of each primer (5 μM, Invitrogen, China), 6 μL of 2 × *Taq* PCR StarMix with loading dye (for PAGE, GenStar, China), and 11.8 μL of ddH_2_O. Thermal cycles were as follows: initial denaturation at 95 °C for 5 min, 35 cycles of 95 °C for 30 s, 59 °C for 30 s, and 72 °C for 40 s, final extension at 72 °C for 5 min, and hold at 10 °C. 400 pairs of SSR primers (Additional file 5: Table S3) were screened for polymorphism in the parents and ten progenies, and 8% acrylamide gels were used for electrophoresis detection. The polymorphic primers were used to characterise the F_1_ segregating population.

### Marker recording

Specific bands were read from top to bottom according to the molecular weights in comparison with a standard DNA marker (50 bp DNA Ladder, Tiangen, China). Clear, high-quality and high-resolution bands with a size of 100–700 bp were selected to improve the recording accuracy and reliability. Polymorphic markers were recorded as 1 or 0 according to their presence or absence, respectively, in the parents and the F_1_ individuals, and vague or missing bands were recorded as 2. All polymorphic markers were divided into three categories (maternal, paternal, and double-simplex markers) according to their presence in the two parents (Table 5).

**Table 5:** Marker types in sweetpotato

	Female parent (Jizishu 1)	Male parent (Longshu 9)	Type of marker
I	Presence / 1	Absence / 0	f (maternal)
II	Absence / 0	Presence / 1	m (paternal)
III	Presence / 1	Presence / 1	ds (double-simplex)

Marker dosage was determined as the segregation ratio of markers (presence:absence) in the mapping population. Four cytological hypotheses proposed by Jones in 1967 were used to classify marker dosages without considering strict tetraploid isolation [33]. Based on the goodness-of-fit to the expected segregation ratios for all markers determined using the Chi-square test, we divided markers into four groups on the basis of their segregation ratios: (1) simplex or single-dose markers exist in one of the parents in the form of a single copy, and the segregation ratio in the progeny is 1:1 (presence:absence), (2) duplex or double-dose markers are present in one of the parents in the form of two copies, and the segregation ratio in the progeny is 4:1 for hexasomic, 5:1 for tetrasomic, or 3:1 for disomic or tetrasomic inheritance, (3) triplex or triple-dose markers are present in one of the parents in three copies, having a ratio of 19:1 (hexasomic), 11:1 (tetradisomic) or 7:1 (disomic); (4) double-simplex markers exist in both parents in a single-dose condition and segregate in a 3:1 ratio in the progeny [5, 8]. According to the Chi-square analysis, if the segregation ratio did not conform to Mendelian segregation, it was considered as a distorted marker.

Marker names were determined by considering the following four points: (1) the polymorphic primer names (e.g., IES87), (2) the corresponding specific band number, usually with a large-molecular-weight band in front (e.g., 05), (3) the type of marker, f, m, or ds (Table 5), (4) the dosage of the marker, s, d, t, or ds, which represented simplex, duplex, triplex or double-simplex, respectively. For example, IES295-1 fs represents the first polymorphic band from SSR primer IES295, and its marker type is a Longshu 9 single marker. For the distorted markers, * and ** as a suffix indicate significant differences at the 0.05 and 0.01 levels, respectively.

### Linkage map construction

The genetic linkage map was constructed using the JoinMap 4.0 software [22] and the pseudo-testcross mapping strategy, as dominant markers that are heterozygous in one parent, and homozygous recessive in the other parent will segregate in the F_1_ generation, resulting in development of two parental linkage maps [34, 35]. Genotype codes for the 300 F_1_ individual plants were recorded using the standard genotype analysis method in JoinMap 4.0. When a band was present only in Jizishu 1, progeny with the same band pattern as that of Jizishu 1 were designated as ‘lm’, while offspring with a different band pattern were marked ‘ll’. When a band was present only in Longshu 9, progeny with the same band pattern as Longshu 9 were designated as ‘np’, otherwise, they were marked ‘nn’. When a band was present in both parents, however, segregated in the progeny, progeny with bands were marked ‘h-’, while those without bands were marked ‘kk’. Only clear bands were recorded, missing and ambiguous bands were represented as ‘-’.

Using the outbreeder full-sib family analysis model, the map was constructed in two steps: (1) single-dose markers were used to construct the framework map of each parent at a logarithm of odds (LOD) of 5.0, (2) duplex and triplex markers were inserted into the framework map to obtain the final genetic linkage map [5, 8]. Linkage groups containing the same duplex or triplex markers were considered homologous and divided into corresponding homologous linkage groups in the same parental map. Linkage groups with the same double-simplex markers in the two maps were considered to be homologous.

Names of linkage groups are primarily composed of three parts: (1) the names of the corresponding parents, JZ1 (Jizishu 1) or L9 (Longshu 9); (2) a number between 1 and 15 (written as 01–15) indicating the sequence number of the homologous linkage group; (3) a number between 1 and 90 (written as 01–90) referring to the sequence number of the linkage group. For example, JZ1 (01.01) indicates that the linkage group belongs to the first linkage group on the Jizishu 1 map, and to the first homologous linkage group. JZ1 (00.66) indicates that the linkage group is the 66th linkage group on the Jizishu 1 map, and is not included in any homologous linkage group.

### Identification of resistance to root rot

The two parents and the 300 F_1_ individuals were planted in the natural disease nursery of Xiong County, Hebei, China (39°06′43″N, 116°14′56″E), the main sweetpotato producing area, on May 16, 2016 and 2017. The experiment was completely randomly arranged, with ridge spacing of 0.85 m and plant spacing of 0.25 m. For each of the F_1_ individuals, five plants were planted, with three repeats. Each parent as the control were planted in each ridge with five. Forty days after planting, as well as in mid-October, the disease index of aboveground and underground was investigated and calculated, respectively.

The final disease index was determined according to the average value of disease index of aboveground and underground. Identification standards for aboveground sweetpotato resistance to root rot is as follows, ‘0’ the plants grow normally, no disease can be seen; ‘1’ the leaves are slightly yellowed, others are normal; ‘2’ the branches are few and short, the leaves are significantly yellowed, and the plant bud or flower; ‘3’ the plant is significantly dwarfed without branching, old leaves fall down from bottom to top; ‘4’ plant has died. Identification standards for underground sweetpotato resistance to root rot are as follows, ‘0’ the fibrous and tuberous roots are normal without any disease spots; ‘1’ a few fibrous roots have turned black (the number of diseased fibrous roots accounts for less than 10% of the total roots), and no disease spots are present on the underground stems, which have no significant effect on tuberous roots formation; ‘2’ a few fibrous roots turn black (the number of diseased fibrous roots accounts for 10–25% of the total roots), and a few diseased spots are apparent on underground stems and tuberous roots, which have a slight effect on tuberous roots formation; ‘3’ nearly half of the fibrous roots have turned black (the number of diseased fibrous roots accounts for 25.1–50.0% of the total roots), with numerous diseased spots are present on underground stems and tuberous roots, which have a significant effect on tuberous roots formation; ‘4’ most of the fibrous roots have turned black (the number of fibrous diseased roots accounts for more than 50% of the total roots), and many large diseased spots are present on the underground stem, without tuberous roots, or the plant has died.

$$ \mathrm{DI}=\frac{\sum \left(\mathrm{A}\ast \mathrm{B}\right)}{\mathrm{C}\ast 4}\ast 100 $$

Where DI represents the disease index of aboveground and underground, respectively, A represents the number of plants at different levels (0, 1, 2, 3 or 4), B represents the corresponding level (0, 1, 2, 3 or 4), C represents the total number of investigated plants, and 4 represents the highest level (4).

### Mapping of QTLs for root rot resistance

The frequency distribution of the 300 F_1_ individual disease index values in 2016 and 2017, as well as the means were determined using IBM SPSS Statistics 24.0 software. Genetic linkage map data, phenotypic data for each year, and the average values were used to map QTLs for root rot resistance using the MapQTL5.0 software [36]. First, interval mapping analysis was used to determine the initial location of the QTL. Second, a multiple QTL model was used to precisely locate the QTL, in which the nearest marker associated with the QTL was selected as the cofactor. In this study, a LOD score of 3.0 was used as the typical threshold value to determine the location of the QTL. QTLs appearing at the same genomic location in the two environments and average data were considered stable QTLs. The linkage maps of QTL for the resistance to root rot were drawn by Map Chart 2.2 [37].

## Supplementary information

**Additional file 1: Figure S1.** SSR linkage maps of Jizishu 1. Each linkage group is identified by a nomenclature that identifies homologous groups (1–15) and linkage groups (1–90). Using this nomenclature, JZ1 (01.01) refers to Jizishu 1 homologous group 1, linkage group 1.The marker name and cumulative map distances (cM) are shown on the right and left sides of the respective linkage group, respectively. The distorted markers are shown with the asterisks * or **, which indicated significant differences at the 0.05 and 0.01 levels, respectively.

**Additional file 2: Figure S2.** SSR linkage maps of Longshu 9. Each linkage group is identified by a nomenclature that identifies homologous groups (1–15) and linkage groups (1–90). Using this nomenclature, L9 (01.01) refers to Longshu 9 homologous group 1, linkage group 1.The marker name and cumulative map distances (cM) are shown on the right and left sides of the respective linkage group, respectively. The distorted markers are shown with the asterisks * or **, which indicated significant differences at the 0.05 and 0.01 levels, respectively.

**Additional file 3: Table S1.** Sequence information of SSR primers for map construction.

**Additional file 4: Table S2.** Corresponding linkage groups among Jizishu 1 and Longshu 9 maps.

**Additional file 5: Table S3.** Sequence information of 400 pairs of SSR primers.

## Data Availability

All data generated or analyzed during this study are included in this published article and its supplementary information files.

## Abbreviations

AFLP: Amplified fragment length polymorphism
EST-SSR: Expressed sequence tag simple sequence repeat
LOD: Logarithm of odds
NGS: Next-generation sequencing
QTL: Quantitative trait locus
RAPD: Random amplified polymorphic DNA
SNP: Single nucleotide polymorphism
SRAP: Sequence-related amplified polymorphism
SSR: Simple sequence repeat
