# Supplementary material for: Identification of QTL for resistance to root rot in sweetpotato (Ipomoea batatas (L.) Lam) with SSR linkage maps
Source: BMC Genomics. 2020 May 15;21:366. doi: 10.1186/s12864-020-06775-9 (PMC7229581; doi:10.1186/s12864-020-06775-9)
Supplement: Supplementary file 1 — Additional file 1: Figure S1. SSR linkage maps of Jizishu 1. Each linkage group is identified by a nomenclature that identifies homologous groups (1–15) and linkage groups (1–90). Using this nomenclature, JZ1 (01.01) refers to Jizishu 1 homologous group 1, linkage group 1.The marker name and cumulative map distances (cM) are shown on the right and left sides of the respective linkage group, respectively. The distorted markers are shown with the asterisks * or **, which indicated significant differences at the 0.05 and 0.01 levels, respectively. [file 12864_2020_6775_MOESM1_ESM.pdf]

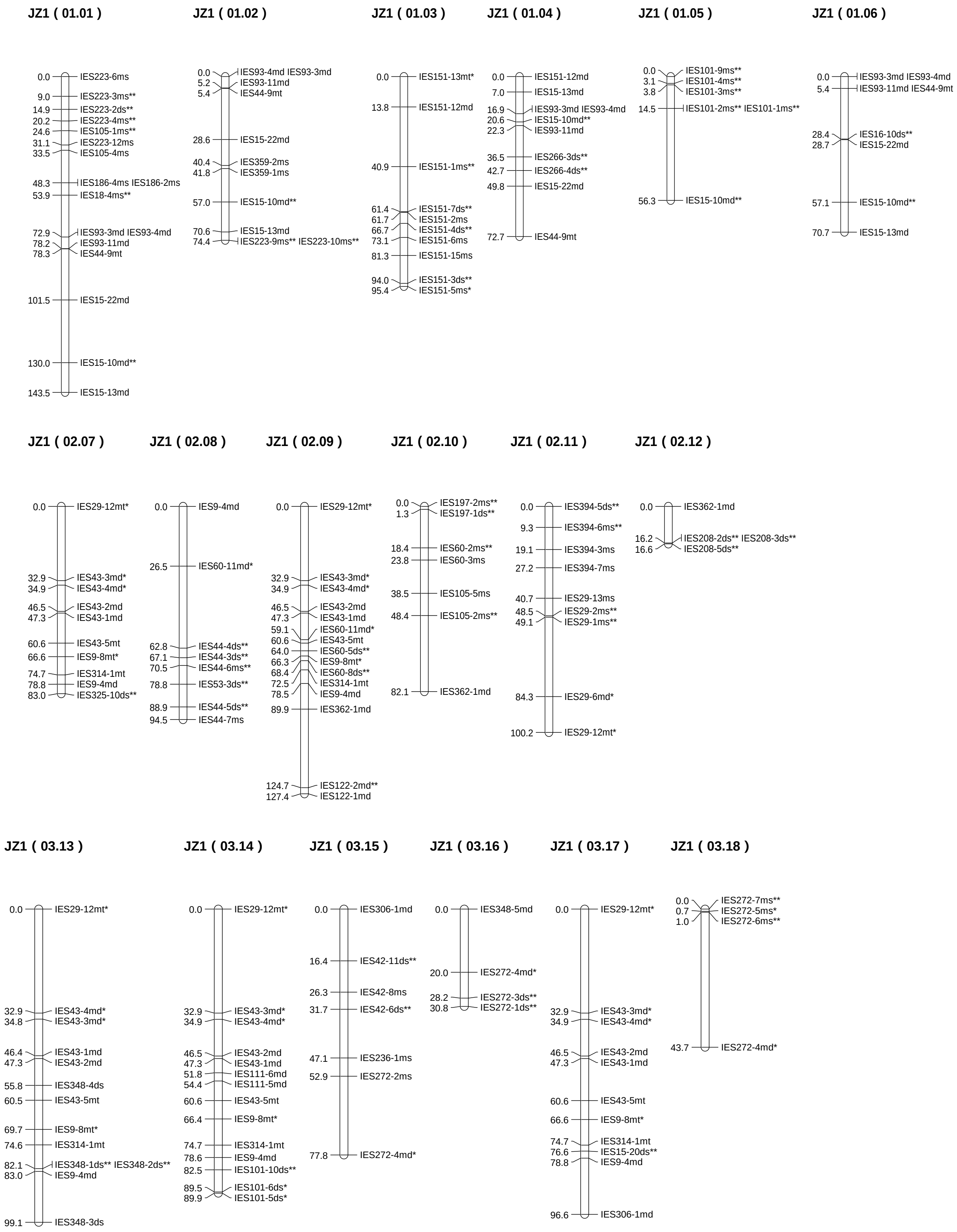
JZ1 (01.01)
0.0 IES223-6ms
9.0 IES223-3ms**
14.9 IES223-2ds**
20.2 IES223-4ms**
24.6 IES105-1ms**
31.1 IES223-12ms
33.5 IES105-4ms
48.3 IES186-4ms IES186-2ms
53.9 IES18-4ms**
72.9 IES93-3md IES93-4md
78.2 IES93-11md
78.3 IES44-9mt
101.5 IES15-22md
130.0 IES15-10md**
143.5 IES15-13md
JZ1 (01.02)
0.0 IES93-4md IES93-3md
5.2 IES93-11md
5.4 IES44-9mt
28.6 IES15-22md
40.4 IES359-2ms
41.8 IES359-1ms
57.0 IES15-10md**
70.6 IES15-13md
74.4 IES223-9ms** IES223-10ms**
JZ1 (01.03)
0.0 IES151-13mt*
13.8 IES151-12md
40.9 IES151-1ms**
61.4 IES151-7ds**
61.7 IES151-2ms
66.7 IES151-4ds**
73.1 IES151-6ms
81.3 IES151-15ms
94.0 IES151-3ds**
95.4 IES151-5ms*
JZ1 (01.04)
0.0 IES151-12md
7.0 IES15-13md
16.9 IES93-3md IES93-4md
20.6 IES15-10md**
22.3 IES93-11md
36.5 IES266-3ds**
42.7 IES266-4ds**
49.8 IES15-22md
72.7 IES44-9mt
JZ1 (01.05)
0.0 IES101-9ms**
3.1 IES101-4ms**
3.8 IES101-3ms**
14.5 IES101-2ms** IES101-1ms**
56.3 IES15-10md**
JZ1 (01.06)
0.0 IES93-3md IES93-4md
5.4 IES93-11md IES44-9mt
28.4 IES16-10ds**
28.7 IES15-22md
57.1 IES15-10md**
70.7 IES15-13md
JZ1 (02.07)
0.0 IES29-12mt*
32.9 IES43-3md*
34.9 IES43-4md*
46.5 IES43-2md
47.3 IES43-1md
60.6 IES43-5mt
66.6 IES9-8mt*
74.7 IES314-1mt
78.8 IES9-4md
83.0 IES325-10ds**
JZ1 (02.08)
0.0 IES9-4md
26.5 IES60-11md*
62.8 IES44-4ds**
67.1 IES44-3ds**
70.5 IES44-6ms**
78.8 IES53-3ds**
88.9 IES44-5ds**
94.5 IES44-7ms
JZ1 (02.09)
0.0 IES29-12mt*
32.9 IES43-3md*
34.9 IES43-4md*
46.5 IES43-2md
47.3 IES43-1md
59.1 IES60-11md*
60.6 IES43-5mt
64.0 IES60-5ds**
66.3 IES9-8mt*
68.4 IES60-8ds**
72.5 IES314-1mt
78.5 IES9-4md
89.9 IES362-1md
124.7 IES122-2md**
127.4 IES122-1md
JZ1 (02.10)
0.0 IES197-2ms**
1.3 IES197-1ds**
18.4 IES60-2ms**
23.8 IES60-3ms
38.5 IES105-5ms
48.4 IES105-2ms**
82.1 IES362-1md
JZ1 (02.11)
0.0 IES394-5ds**
9.3 IES394-6ms**
19.1 IES394-3ms
27.2 IES394-7ms
40.7 IES29-13ms
48.5 IES29-2ms**
49.1 IES29-1ms**
84.3 IES29-6md*
100.2 IES29-12mt*
JZ1 (02.12)
0.0 IES362-1md
16.2 IES208-2ds** IES208-3ds**
16.6 IES208-5ds**
JZ1 (03.13)
0.0 IES29-12mt*
32.9 IES43-4md*
34.8 IES43-3md*
46.4 IES43-1md
47.3 IES43-2md
55.8 IES348-4ds
60.5 IES43-5mt
69.7 IES9-8mt*
74.6 IES314-1mt
82.1 IES348-1ds** IES348-2ds**
83.0 IES9-4md
99.1 IES348-3ds
JZ1 (03.14)
0.0 IES29-12mt*
32.9 IES43-3md*
34.9 IES43-4md*
46.5 IES43-2md
47.3 IES43-1md
51.8 IES111-6md
54.4 IES111-5md
60.6 IES43-5mt
66.4 IES9-8mt*
74.7 IES314-1mt
78.6 IES9-4md
82.5 IES101-10ds**
89.5 IES101-6ds*
89.9 IES101-5ds*
JZ1 (03.15)
0.0 IES306-1md
16.4 IES42-11ds**
26.3 IES42-8ms
31.7 IES42-6ds**
47.1 IES236-1ms
52.9 IES272-2ms
77.8 IES272-4md*
JZ1 (03.16)
0.0 IES348-5md
20.0 IES272-4md*
28.2 IES272-3ds**
30.8 IES272-1ds**
JZ1 (03.17)
0.0 IES29-12mt*
32.9 IES43-3md*
34.9 IES43-4md*
46.5 IES43-2md
47.3 IES43-1md
60.6 IES43-5mt
66.6 IES9-8mt*
74.7 IES314-1mt
76.6 IES15-20ds**
78.8 IES9-4md
96.6 IES306-1md
JZ1 (03.18)
0.0 IES272-7ms**
0.7 IES272-5ms*
1.0 IES272-6ms**
43.7 IES272-4md*

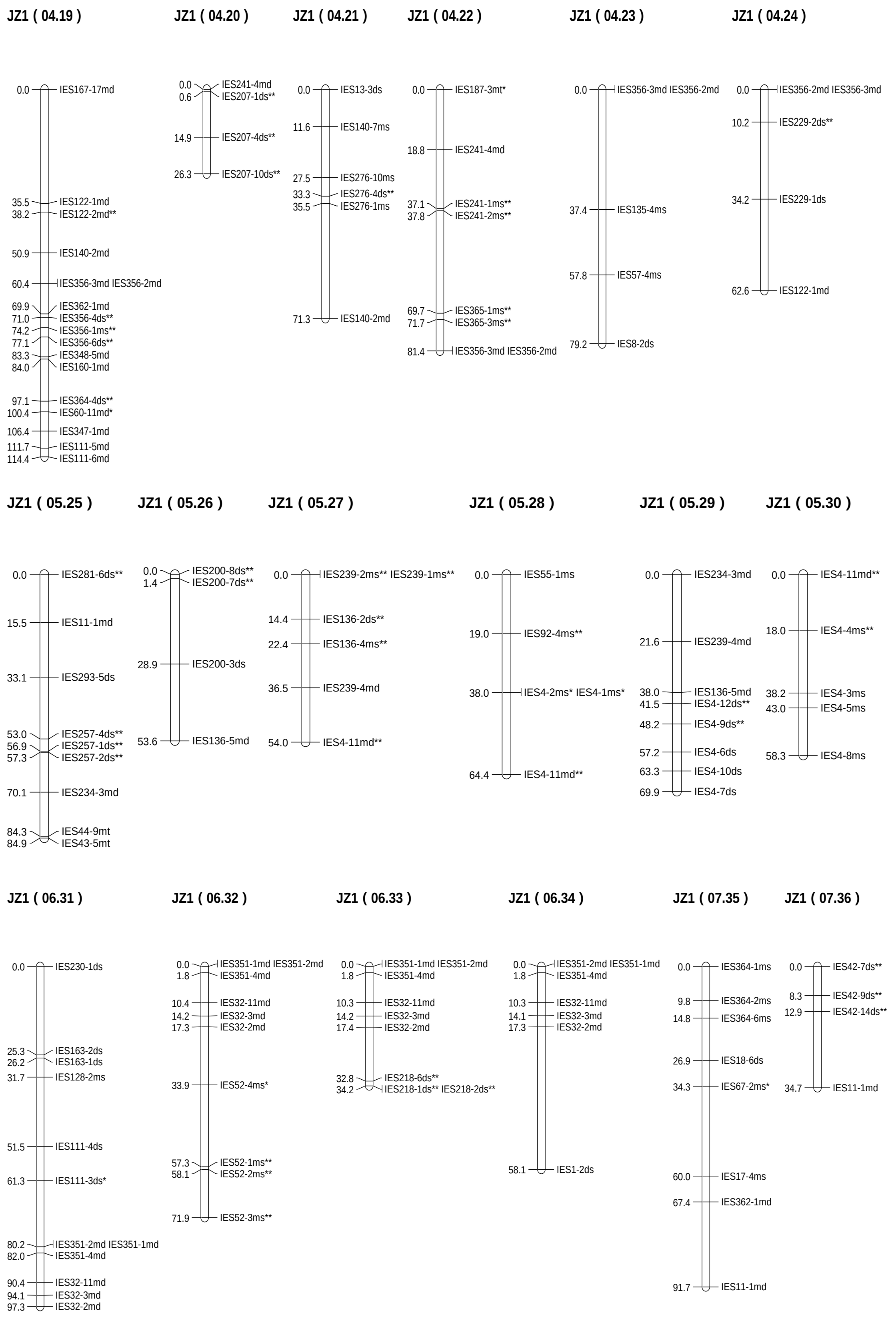
JZ1 (04.19)
0.0 IES167-17md
35.5 IES122-1md
38.2 IES122-2md**
50.9 IES140-2md
60.4 IES356-3md IES356-2md
69.9 IES362-1md
71.0 IES356-4ds**
74.2 IES356-1ms**
77.1 IES356-6ds**
83.3 IES348-5md
84.0 IES160-1md
97.1 IES364-4ds**
100.4 IES60-11md*
106.4 IES347-1md
111.7 IES111-5md
114.4 IES111-6md
JZ1 (04.20)
0.0 IES241-4md
0.6 IES207-1ds**
14.9 IES207-4ds**
26.3 IES207-10ds**
JZ1 (04.21)
0.0 IES13-3ds
11.6 IES140-7ms
27.5 IES276-10ms
33.3 IES276-4ds**
35.5 IES276-1ms
71.3 IES140-2md
JZ1 (04.22)
0.0 IES187-3mt*
18.8 IES241-4md
37.1 IES241-1ms**
37.8 IES241-2ms**
69.7 IES365-1ms**
71.7 IES365-3ms**
81.4 IES356-3md IES356-2md
JZ1 (04.23)
0.0 IES356-3md IES356-2md
37.4 IES135-4ms
57.8 IES57-4ms
79.2 IES8-2ds
JZ1 (04.24)
0.0 IES356-2md IES356-3md
10.2 IES229-2ds**
34.2 IES229-1ds
62.6 IES122-1md
JZ1 (05.25)
0.0 IES281-6ds**
15.5 IES11-1md
33.1 IES293-5ds
53.0 IES257-4ds**
56.9 IES257-1ds**
57.3 IES257-2ds**
70.1 IES234-3md
84.3 IES44-9mt
84.9 IES43-5mt
JZ1 (05.26)
0.0 IES200-8ds**
1.4 IES200-7ds**
28.9 IES200-3ds
53.6 IES136-5md
JZ1 (05.27)
0.0 IES239-2ms** IES239-1ms**
14.4 IES136-2ds**
22.4 IES136-4ms**
36.5 IES239-4md
54.0 IES4-11md**
JZ1 (05.28)
0.0 IES55-1ms
19.0 IES92-4ms**
38.0 IES4-2ms* IES4-1ms*
64.4 IES4-11md**
JZ1 (05.29)
0.0 IES234-3md
21.6 IES239-4md
38.0 IES136-5md
41.5 IES4-12ds**
48.2 IES4-9ds**
57.2 IES4-6ds
63.3 IES4-10ds
69.9 IES4-7ds
JZ1 (05.30)
0.0 IES4-11md**
18.0 IES4-4ms**
38.2 IES4-3ms
43.0 IES4-5ms
58.3 IES4-8ms
JZ1 (06.31)
0.0 IES230-1ds
25.3 IES163-2ds
26.2 IES163-1ds
31.7 IES128-2ms
51.5 IES111-4ds
61.3 IES111-3ds*
80.2 IES351-2md IES351-1md
82.0 IES351-4md
90.4 IES32-11md
94.1 IES32-3md
97.3 IES32-2md
JZ1 (06.32)
0.0 IES351-1md IES351-2md
1.8 IES351-4md
10.4 IES32-11md
14.2 IES32-3md
17.3 IES32-2md
33.9 IES52-4ms*
57.3 IES52-1ms**
58.1 IES52-2ms**
71.9 IES52-3ms**
JZ1 (06.33)
0.0 IES351-1md IES351-2md
1.8 IES351-4md
10.3 IES32-11md
14.2 IES32-3md
17.4 IES32-2md
32.8 IES218-6ds**
34.2 IES218-1ds** IES218-2ds**
JZ1 (06.34)
0.0 IES351-2md IES351-1md
1.8 IES351-4md
10.3 IES32-11md
14.1 IES32-3md
17.3 IES32-2md
58.1 IES1-2ds
JZ1 (07.35)
0.0 IES364-1ms
9.8 IES364-2ms
14.8 IES364-6ms
26.9 IES18-6ds
34.3 IES67-2ms*
60.0 IES17-4ms
67.4 IES362-1md
91.7 IES11-1md
JZ1 (07.36)
0.0 IES42-7ds**
8.3 IES42-9ds**
12.9 IES42-14ds**
34.7 IES11-1md

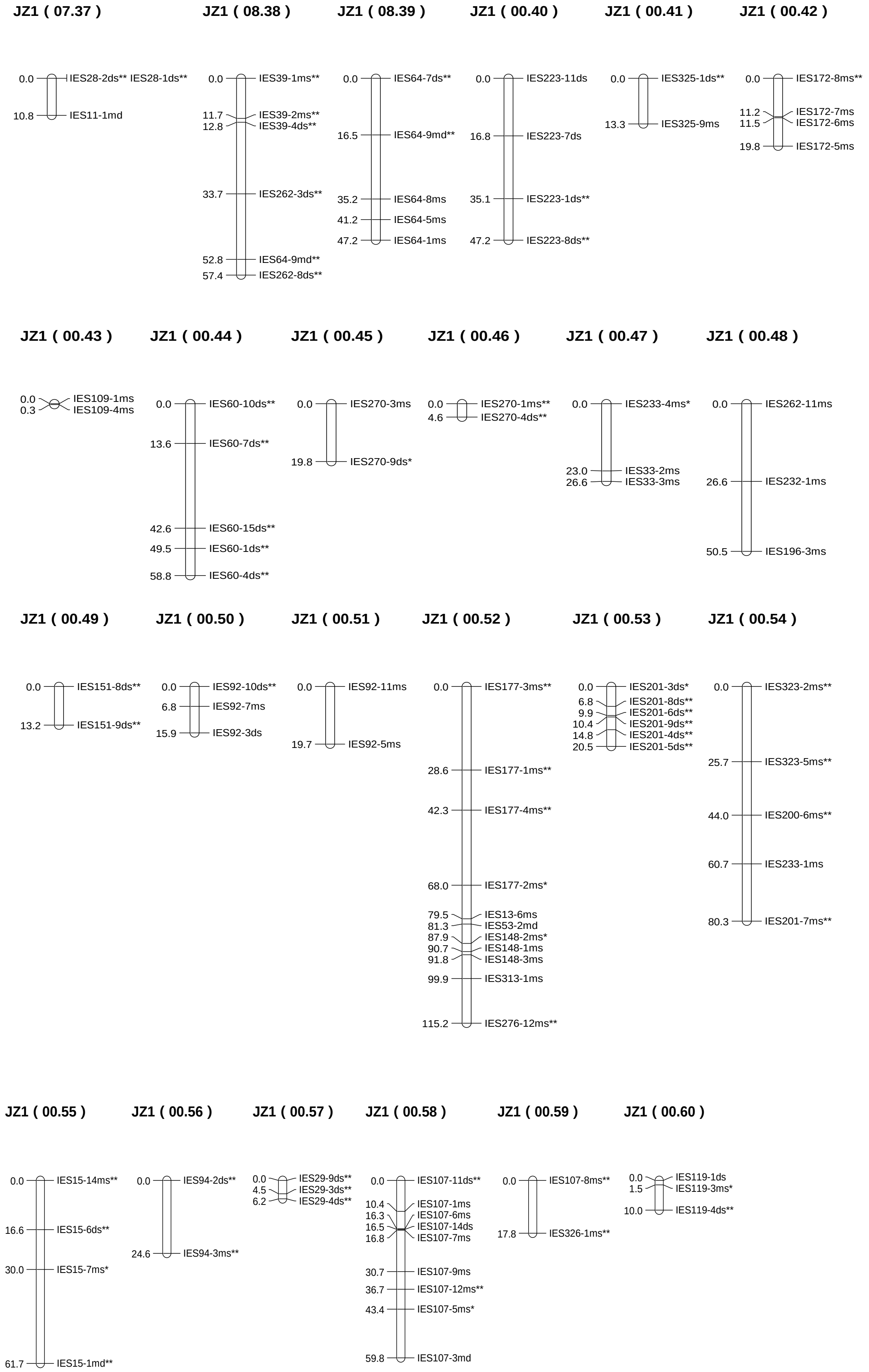

JZ1 (07.37)
0.0 IES28-2ds** IES28-1ds**
10.8 IES11-1md
JZ1 (08.38)
0.0 IES39-1ms**
11.7 IES39-2ms**
12.8 IES39-4ds**
33.7 IES262-3ds**
52.8 IES64-9md**
57.4 IES262-8ds**
JZ1 (08.39)
0.0 IES64-7ds**
16.5 IES64-9md**
35.2 IES64-8ms
41.2 IES64-5ms
47.2 IES64-1ms
JZ1 (00.40)
0.0 IES223-11ds
16.8 IES223-7ds
35.1 IES223-1ds**
47.2 IES223-8ds**
JZ1 (00.41)
0.0 IES325-1ds**
13.3 IES325-9ms
JZ1 (00.42)
0.0 IES172-8ms**
11.2 IES172-7ms
11.5 IES172-6ms
19.8 IES172-5ms
JZ1 (00.43)
0.0 IES109-1ms
0.3 IES109-4ms
JZ1 (00.44)
0.0 IES60-10ds**
13.6 IES60-7ds**
42.6 IES60-15ds**
49.5 IES60-1ds**
58.8 IES60-4ds**
JZ1 (00.45)
0.0 IES270-3ms
19.8 IES270-9ds*
JZ1 (00.46)
0.0 IES270-1ms**
4.6 IES270-4ds**
JZ1 (00.47)
0.0 IES233-4ms*
23.0 IES33-2ms
26.6 IES33-3ms
JZ1 (00.48)
0.0 IES262-11ms
26.6 IES232-1ms
50.5 IES196-3ms
JZ1 (00.49)
0.0 IES151-8ds**
13.2 IES151-9ds**
JZ1 (00.50)
0.0 IES92-10ds**
6.8 IES92-7ms
15.9 IES92-3ds
JZ1 (00.51)
0.0 IES92-11ms
19.7 IES92-5ms
JZ1 (00.52)
0.0 IES177-3ms**
28.6 IES177-1ms**
42.3 IES177-4ms**
68.0 IES177-2ms*
79.5 IES13-6ms
81.3 IES53-2md
87.9 IES148-2ms*
90.7 IES148-1ms
91.8 IES148-3ms
99.9 IES313-1ms
115.2 IES276-12ms**
JZ1 (00.53)
0.0 IES201-3ds*
6.8 IES201-8ds**
9.9 IES201-6ds**
10.4 IES201-9ds**
14.8 IES201-4ds**
20.5 IES201-5ds**
JZ1 (00.54)
0.0 IES323-2ms**
25.7 IES323-5ms**
44.0 IES200-6ms**
60.7 IES233-1ms
80.3 IES201-7ms**
JZ1 (00.55)
0.0 IES15-14ms**
16.6 IES15-6ds**
30.0 IES15-7ms*
61.7 IES15-1md**
JZ1 (00.56)
0.0 IES94-2ds**
24.6 IES94-3ms**
JZ1 (00.57)
0.0 IES29-9ds**
4.5 IES29-3ds**
6.2 IES29-4ds**
JZ1 (00.58)
0.0 IES107-11ds**
10.4 IES107-1ms
16.3 IES107-6ms
16.5 IES107-14ds
16.8 IES107-7ms
30.7 IES107-9ms
36.7 IES107-12ms**
43.4 IES107-5ms*
59.8 IES107-3md
JZ1 (00.59)
0.0 IES107-8ms**
17.8 IES326-1ms**
JZ1 (00.60)
0.0 IES119-1ds
1.5 IES119-3ms*
10.0 IES119-4ds**

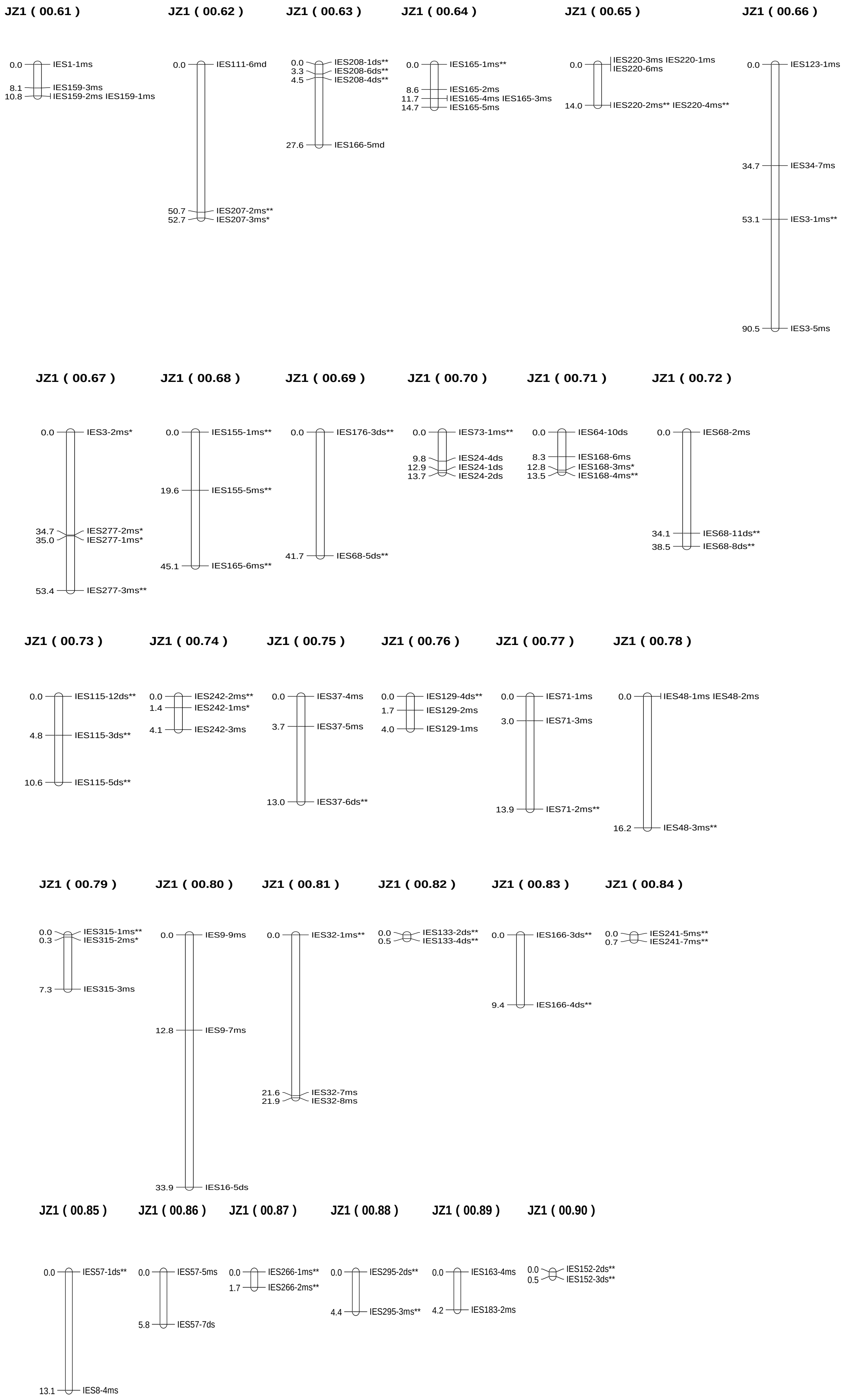
JZ1 (00.61)
0.0 IES1-1ms
8.1 IES159-3ms
10.8 IES159-2ms IES159-1ms
JZ1 (00.62)
0.0 IES111-6md
50.7 IES207-2ms**
52.7 IES207-3ms*
JZ1 (00.63)
0.0 IES208-1ds**
3.3 IES208-6ds**
4.5 IES208-4ds**
27.6 IES166-5md
JZ1 (00.64)
0.0 IES165-1ms**
8.6 IES165-2ms
11.7 IES165-4ms IES165-3ms
14.7 IES165-5ms
JZ1 (00.65)
0.0 IES220-3ms IES220-1ms IES220-6ms
14.0 IES220-2ms** IES220-4ms**
JZ1 (00.66)
0.0 IES123-1ms
34.7 IES34-7ms
53.1 IES3-1ms**
90.5 IES3-5ms
JZ1 (00.67)
0.0 IES3-2ms*
34.7 IES277-2ms*
35.0 IES277-1ms*
53.4 IES277-3ms**
JZ1 (00.68)
0.0 IES155-1ms**
19.6 IES155-5ms**
45.1 IES165-6ms**
JZ1 (00.69)
0.0 IES176-3ds**
41.7 IES68-5ds**
JZ1 (00.70)
0.0 IES73-1ms**
9.8 IES24-4ds
12.9 IES24-1ds
13.7 IES24-2ds
JZ1 (00.71)
0.0 IES64-10ds
8.3 IES168-6ms
12.8 IES168-3ms*
13.5 IES168-4ms**
JZ1 (00.72)
0.0 IES68-2ms
34.1 IES68-11ds**
38.5 IES68-8ds**
JZ1 (00.73)
0.0 IES115-12ds**
4.8 IES115-3ds**
10.6 IES115-5ds**
JZ1 (00.74)
0.0 IES242-2ms**
1.4 IES242-1ms*
4.1 IES242-3ms
JZ1 (00.75)
0.0 IES37-4ms
3.7 IES37-5ms
13.0 IES37-6ds**
JZ1 (00.76)
0.0 IES129-4ds**
1.7 IES129-2ms
4.0 IES129-1ms
JZ1 (00.77)
0.0 IES71-1ms
3.0 IES71-3ms
13.9 IES71-2ms**
JZ1 (00.78)
0.0 IES48-1ms IES48-2ms
16.2 IES48-3ms**
JZ1 (00.79)
0.0 IES315-1ms**
0.3 IES315-2ms*
7.3 IES315-3ms
JZ1 (00.80)
0.0 IES9-9ms
12.8 IES9-7ms
33.9 IES16-5ds
JZ1 (00.81)
0.0 IES32-1ms**
21.6 IES32-7ms
21.9 IES32-8ms
JZ1 (00.82)
0.0 IES133-2ds**
0.5 IES133-4ds**
JZ1 (00.83)
0.0 IES166-3ds**
9.4 IES166-4ds**
JZ1 (00.84)
0.0 IES241-5ms**
0.7 IES241-7ms**
JZ1 (00.85)
0.0 IES57-1ds**
13.1 IES8-4ms
JZ1 (00.86)
0.0 IES57-5ms
5.8 IES57-7ds
JZ1 (00.87)
0.0 IES266-1ms**
1.7 IES266-2ms**
JZ1 (00.88)
0.0 IES295-2ds**
4.4 IES295-3ms**
JZ1 (00.89)
0.0 IES163-4ms
4.2 IES183-2ms
JZ1 (00.90)
0.0 IES152-2ds**
0.5 IES152-3ds**
