# Supplementary material for: Identification of QTL for resistance to root rot in sweetpotato (Ipomoea batatas (L.) Lam) with SSR linkage maps
Source: BMC Genomics. 2020 May 15;21:366. doi: 10.1186/s12864-020-06775-9 (PMC7229581; doi:10.1186/s12864-020-06775-9)
Supplement: Supplementary file 2 — Additional file 2: Figure S2. SSR linkage maps of Longshu 9. Each linkage group is identified by a nomenclature that identifies homologous groups (1–15) and linkage groups (1–90). Using this nomenclature, L9 (01.01) refers to Longshu 9 homologous group 1, linkage group 1.The marker name and cumulative map distances (cM) are shown on the right and left sides of the respective linkage group, respectively. The distorted markers are shown with the asterisks * or **, which indicated significant differences at the 0.05 and 0.01 levels, respectively. [file 12864_2020_6775_MOESM2_ESM.pdf]

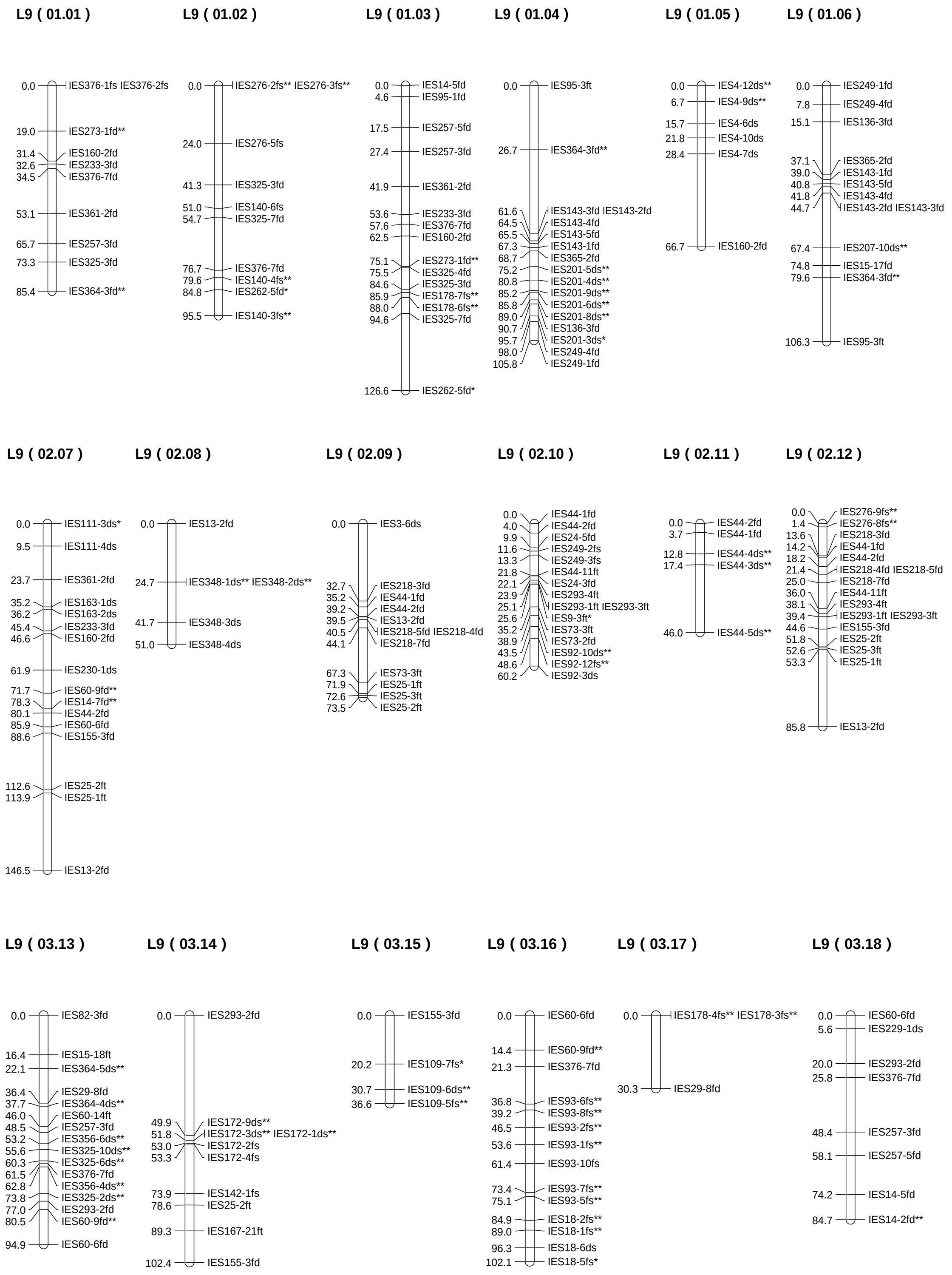
L9 (01.01)
0.0 IES376-1fs IES376-2fs
19.0 IES273-1fd**
31.4 IES160-2fd
32.6 IES233-3fd
34.5 IES376-7fd
53.1 IES361-2fd
65.7 IES257-3fd
73.3 IES325-3fd
85.4 IES364-3fd**
L9 (01.02)
0.0 IES276-2fs** IES276-3fs**
24.0 IES276-5fs
41.3 IES325-3fd
51.0 IES140-6fs
54.7 IES325-7fd
76.7 IES376-7fd
79.6 IES140-4fs**
84.8 IES262-5fd*
95.5 IES140-3fs**
L9 (01.03)
0.0 IES14-5fd
4.6 IES95-1fd
17.5 IES257-5fd
27.4 IES257-3fd
41.9 IES361-2fd
53.6 IES233-3fd
57.6 IES376-7fd
62.5 IES160-2fd
75.1 IES273-1fd**
75.5 IES325-4fd
84.6 IES325-3fd
85.9 IES178-7fs**
88.0 IES178-6fs**
94.6 IES325-7fd
126.6 IES262-5fd*
L9 (01.04)
0.0 IES95-3ft
26.7 IES364-3fd**
61.6 IES143-3fd IES143-2fd
64.5 IES143-4fd
65.5 IES143-5fd
67.3 IES143-1fd
68.7 IES365-2fd
75.2 IES201-5ds**
80.8 IES201-4ds**
85.2 IES201-9ds**
85.8 IES201-6ds**
89.0 IES201-8ds**
90.7 IES136-3fd
95.7 IES201-3ds*
98.0 IES249-4fd
105.8 IES249-1fd
L9 (01.05)
0.0 IES4-12ds**
6.7 IES4-9ds**
15.7 IES4-6ds
21.8 IES4-10ds
28.4 IES4-7ds
66.7 IES160-2fd
L9 (01.06)
0.0 IES249-1fd
7.8 IES249-4fd
15.1 IES136-3fd
37.1 IES365-2fd
39.0 IES143-1fd
40.8 IES143-5fd
41.8 IES143-4fd
44.7 IES143-2fd IES143-3fd
67.4 IES207-10ds**
74.8 IES15-17fd
79.6 IES364-3fd**
106.3 IES95-3ft
L9 (02.07)
0.0 IES111-3ds*
9.5 IES111-4ds
23.7 IES361-2fd
35.2 IES163-1ds
36.2 IES163-2ds
45.4 IES233-3fd
46.6 IES160-2fd
61.9 IES230-1ds
71.7 IES60-9fd**
78.3 IES14-7fd**
80.1 IES44-2fd
85.9 IES60-6fd
88.6 IES155-3fd
112.6 IES25-2ft
113.9 IES25-1ft
146.5 IES13-2fd
L9 (02.08)
0.0 IES13-2fd
24.7 IES348-1ds** IES348-2ds**
41.7 IES348-3ds
51.0 IES348-4ds
L9 (02.09)
0.0 IES3-6ds
32.7 IES218-3fd
35.2 IES44-1fd
39.2 IES44-2fd
39.5 IES13-2fd
40.5 IES218-5fd IES218-4fd
44.1 IES218-7fd
67.3 IES73-3ft
71.9 IES25-1ft
72.6 IES25-3ft
73.5 IES25-2ft
L9 (02.10)
0.0 IES44-1fd
4.0 IES44-2fd
9.9 IES24-5fd
11.6 IES249-2fs
13.3 IES249-3fs
21.8 IES44-11ft
22.1 IES24-3fd
23.9 IES293-4ft
25.1 IES293-1ft IES293-3ft
25.6 IES9-3ft*
35.2 IES73-3ft
38.9 IES73-2fd
43.5 IES92-10ds**
48.6 IES92-12fs**
60.2 IES92-3ds
L9 (02.11)
0.0 IES44-2fd
3.7 IES44-1fd
12.8 IES44-4ds**
17.4 IES44-3ds**
46.0 IES44-5ds**
L9 (02.12)
0.0 IES276-9fs**
1.4 IES276-8fs**
13.6 IES218-3fd
14.2 IES44-1fd
18.2 IES44-2fd
21.4 IES218-4fd IES218-5fd
25.0 IES218-7fd
36.0 IES44-11ft
38.1 IES293-4ft
39.4 IES293-1ft IES293-3ft
44.6 IES155-3fd
51.8 IES25-2ft
52.6 IES25-3ft
53.3 IES25-1ft
85.8 IES13-2fd
L9 (03.13)
0.0 IES82-3fd
16.4 IES15-18ft
22.1 IES364-5ds**
36.4 IES29-8fd
37.7 IES364-4ds**
46.0 IES60-14ft
48.5 IES257-3fd
53.2 IES356-6ds**
55.6 IES325-10ds**
60.3 IES325-6ds**
61.5 IES376-7fd
62.8 IES356-4ds**
73.8 IES325-2ds**
77.0 IES293-2fd
80.5 IES60-9fd**
94.9 IES60-6fd
L9 (03.14)
0.0 IES293-2fd
49.9 IES172-9ds**
51.8 IES172-3ds** IES172-1ds**
53.0 IES172-2fs
53.3 IES172-4fs
73.9 IES142-1fs
78.6 IES25-2ft
89.3 IES167-21ft
102.4 IES155-3fd
L9 (03.15)
0.0 IES155-3fd
20.2 IES109-7fs*
30.7 IES109-6ds**
36.6 IES109-5fs**
L9 (03.16)
0.0 IES60-6fd
14.4 IES60-9fd**
21.3 IES376-7fd
36.8 IES93-6fs**
39.2 IES93-8fs**
46.5 IES93-2fs**
53.6 IES93-1fs**
61.4 IES93-10fs
73.4 IES93-7fs**
75.1 IES93-5fs**
84.9 IES18-2fs**
89.0 IES18-1fs**
96.3 IES18-6ds
102.1 IES18-5fs*
L9 (03.17)
0.0 IES178-4fs** IES178-3fs**
30.3 IES29-8fd
L9 (03.18)
0.0 IES60-6fd
5.6 IES229-1ds
20.0 IES293-2fd
25.8 IES376-7fd
48.4 IES257-3fd
58.1 IES257-5fd
74.2 IES14-5fd
84.7 IES14-2fd**

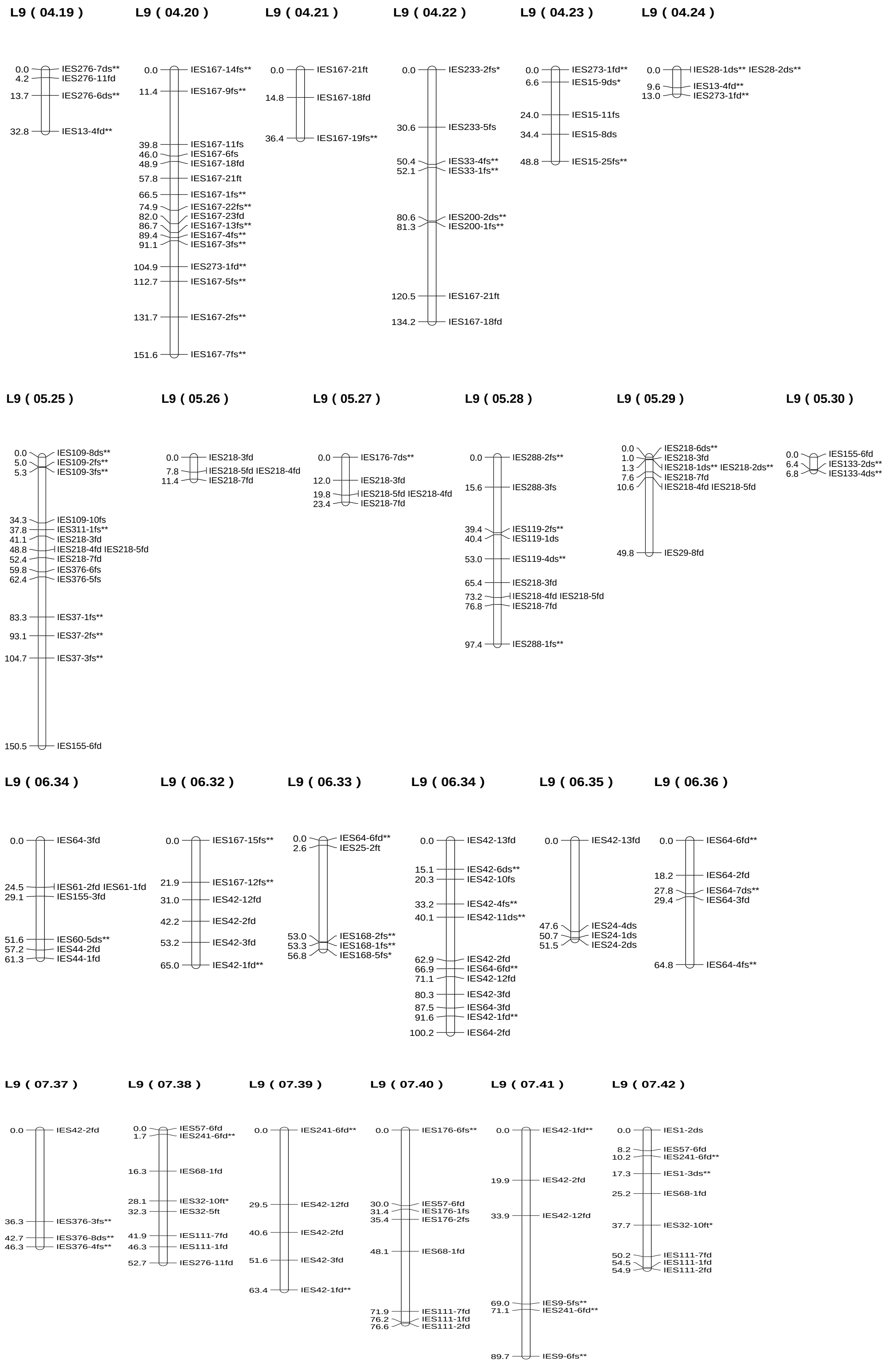
L9 (04.19)
0.0 IES276-7ds**
4.2 IES276-11fd
13.7 IES276-6ds**
32.8 IES13-4fd**
L9 (04.20)
0.0 IES167-14fs**
11.4 IES167-9fs**
39.8 IES167-11fs
46.0 IES167-6fs
48.9 IES167-18fd
57.8 IES167-21ft
66.5 IES167-1fs**
74.9 IES167-22fs**
82.0 IES167-23fd
86.7 IES167-13fs**
89.4 IES167-4fs**
91.1 IES167-3fs**
104.9 IES273-1fd**
112.7 IES167-5fs**
131.7 IES167-2fs**
151.6 IES167-7fs**
L9 (04.21)
0.0 IES167-21ft
14.8 IES167-18fd
36.4 IES167-19fs**
L9 (04.22)
0.0 IES233-2fs*
30.6 IES233-5fs
50.4 IES33-4fs**
52.1 IES33-1fs**
80.6 IES200-2ds**
81.3 IES200-1fs**
120.5 IES167-21ft
134.2 IES167-18fd
L9 (04.23)
0.0 IES273-1fd**
6.6 IES15-9ds*
24.0 IES15-11fs
34.4 IES15-8ds
48.8 IES15-25fs**
L9 (04.24)
0.0 IES28-1ds** IES28-2ds**
9.6 IES13-4fd**
13.0 IES273-1fd**
L9 (05.25)
0.0 IES109-8ds**
5.0 IES109-2fs**
5.3 IES109-3fs**
34.3 IES109-10fs
37.8 IES311-1fs**
41.1 IES218-3fd
48.8 IES218-4fd IES218-5fd
52.4 IES218-7fd
59.8 IES376-6fs
62.4 IES376-5fs
83.3 IES37-1fs**
93.1 IES37-2fs**
104.7 IES37-3fs**
150.5 IES155-6fd
L9 (05.26)
0.0 IES218-3fd
7.8 IES218-5fd IES218-4fd
11.4 IES218-7fd
L9 (05.27)
0.0 IES176-7ds**
12.0 IES218-3fd
19.8 IES218-5fd IES218-4fd
23.4 IES218-7fd
L9 (05.28)
0.0 IES288-2fs**
15.6 IES288-3fs
39.4 IES119-2fs**
40.4 IES119-1ds
53.0 IES119-4ds**
65.4 IES218-3fd
73.2 IES218-4fd IES218-5fd
76.8 IES218-7fd
97.4 IES288-1fs**
L9 (05.29)
0.0 IES218-6ds**
1.0 IES218-3fd
1.3 IES218-1ds** IES218-2ds**
7.6 IES218-7fd
10.6 IES218-4fd IES218-5fd
49.8 IES29-8fd
L9 (05.30)
0.0 IES155-6fd
6.4 IES133-2ds**
6.8 IES133-4ds**
L9 (06.34)
0.0 IES64-3fd
24.5 IES61-2fd IES61-1fd
29.1 IES155-3fd
51.6 IES60-5ds**
57.2 IES44-2fd
61.3 IES44-1fd
L9 (06.32)
0.0 IES167-15fs**
21.9 IES167-12fs**
31.0 IES42-12fd
42.2 IES42-2fd
53.2 IES42-3fd
65.0 IES42-1fd**
L9 (06.33)
0.0 IES64-6fd**
2.6 IES25-2ft
53.0 IES168-2fs**
53.3 IES168-1fs**
56.8 IES168-5fs*
L9 (06.34)
0.0 IES42-13fd
15.1 IES42-6ds**
20.3 IES42-10fs
33.2 IES42-4fs**
40.1 IES42-11ds**
62.9 IES42-2fd
66.9 IES64-6fd**
71.1 IES42-12fd
80.3 IES42-3fd
87.5 IES64-3fd
91.6 IES42-1fd**
100.2 IES64-2fd
L9 (06.35)
0.0 IES42-13fd
47.6 IES24-4ds
50.7 IES24-1ds
51.5 IES24-2ds
L9 (06.36)
0.0 IES64-6fd**
18.2 IES64-2fd
27.8 IES64-7ds**
29.4 IES64-3fd
64.8 IES64-4fs**
L9 (07.37)
0.0 IES42-2fd
36.3 IES376-3fs**
42.7 IES376-8ds**
46.3 IES376-4fs**
L9 (07.38)
0.0 IES57-6fd
1.7 IES241-6fd**
16.3 IES68-1fd
28.1 IES32-10ft*
32.3 IES32-5ft
41.9 IES111-7fd
46.3 IES111-1fd
52.7 IES276-11fd
L9 (07.39)
0.0 IES241-6fd**
29.5 IES42-12fd
40.6 IES42-2fd
51.6 IES42-3fd
63.4 IES42-1fd**
L9 (07.40)
0.0 IES176-6fs**
30.0 IES57-6fd
31.4 IES176-1fs
35.4 IES176-2fs
48.1 IES68-1fd
71.9 IES111-7fd
76.2 IES111-1fd
76.6 IES111-2fd
L9 (07.41)
0.0 IES42-1fd**
19.9 IES42-2fd
33.9 IES42-12fd
69.0 IES9-5fs**
71.1 IES241-6fd**
89.7 IES9-6fs**
L9 (07.42)
0.0 IES1-2ds
8.2 IES57-6fd
10.2 IES241-6fd**
17.3 IES1-3ds**
25.2 IES68-1fd
37.7 IES32-10ft*
50.2 IES111-7fd
54.5 IES111-1fd
54.9 IES111-2fd

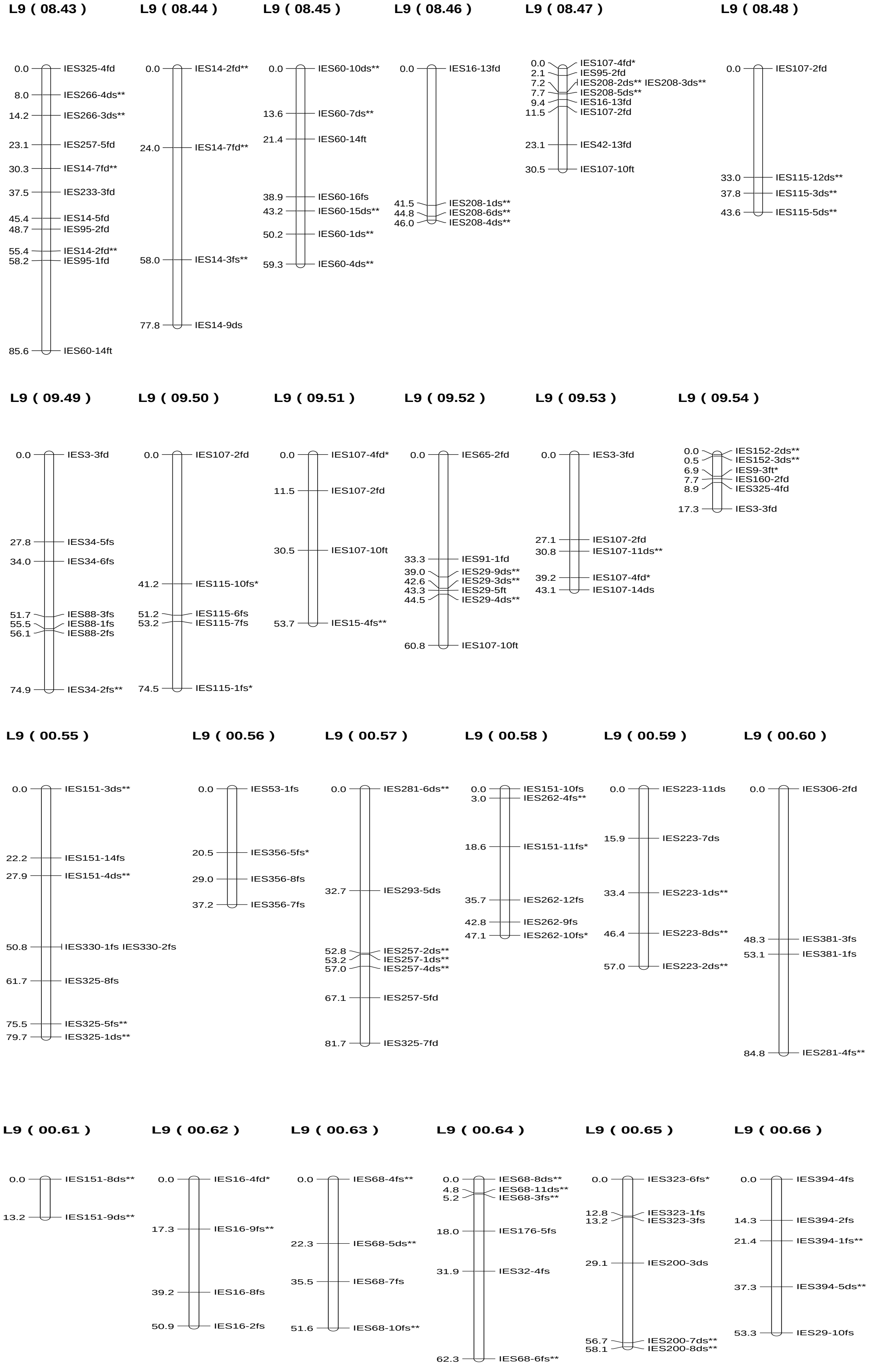
L9 (08.43)
0.0 IES325-4fd
8.0 IES266-4ds**
14.2 IES266-3ds**
23.1 IES257-5fd
30.3 IES14-7fd**
37.5 IES233-3fd
45.4 IES14-5fd
48.7 IES95-2fd
55.4 IES14-2fd**
58.2 IES95-1fd
85.6 IES60-14ft
L9 (08.44)
0.0 IES14-2fd**
24.0 IES14-7fd**
58.0 IES14-3fs**
77.8 IES14-9ds
L9 (08.45)
0.0 IES60-10ds**
13.6 IES60-7ds**
21.4 IES60-14ft
38.9 IES60-16fs
43.2 IES60-15ds**
50.2 IES60-1ds**
59.3 IES60-4ds**
L9 (08.46)
0.0 IES16-13fd
41.5 IES208-1ds**
44.8 IES208-6ds**
46.0 IES208-4ds**
L9 (08.47)
0.0 IES107-4fd*
2.1 IES95-2fd
7.2 IES208-2ds** IES208-3ds**
7.7 IES208-5ds**
9.4 IES16-13fd
11.5 IES107-2fd
23.1 IES42-13fd
30.5 IES107-10ft
L9 (08.48)
0.0 IES107-2fd
33.0 IES115-12ds**
37.8 IES115-3ds**
43.6 IES115-5ds**
L9 (09.49)
0.0 IES3-3fd
27.8 IES34-5fs
34.0 IES34-6fs
51.7 IES88-3fs
55.5 IES88-1fs
56.1 IES88-2fs
74.9 IES34-2fs**
L9 (09.50)
0.0 IES107-2fd
41.2 IES115-10fs*
51.2 IES115-6fs
53.2 IES115-7fs
74.5 IES115-1fs*
L9 (09.51)
0.0 IES107-4fd*
11.5 IES107-2fd
30.5 IES107-10ft
53.7 IES15-4fs**
L9 (09.52)
0.0 IES65-2fd
33.3 IES91-1fd
39.0 IES29-9ds**
42.6 IES29-3ds**
43.3 IES29-5ft
44.5 IES29-4ds**
60.8 IES107-10ft
L9 (09.53)
0.0 IES3-3fd
27.1 IES107-2fd
30.8 IES107-11ds**
39.2 IES107-4fd*
43.1 IES107-14ds
L9 (09.54)
0.0 IES152-2ds**
0.5 IES152-3ds**
6.9 IES9-3ft*
7.7 IES160-2fd
8.9 IES325-4fd
17.3 IES3-3fd
L9 (00.55)
0.0 IES151-3ds**
22.2 IES151-14fs
27.9 IES151-4ds**
50.8 IES330-1fs IES330-2fs
61.7 IES325-8fs
75.5 IES325-5fs**
79.7 IES325-1ds**
L9 (00.56)
0.0 IES53-1fs
20.5 IES356-5fs*
29.0 IES356-8fs
37.2 IES356-7fs
L9 (00.57)
0.0 IES281-6ds**
32.7 IES293-5ds
52.8 IES257-2ds**
53.2 IES257-1ds**
57.0 IES257-4ds**
67.1 IES257-5fd
81.7 IES325-7fd
L9 (00.58)
0.0 IES151-10fs
3.0 IES262-4fs**
18.6 IES151-11fs*
35.7 IES262-12fs
42.8 IES262-9fs
47.1 IES262-10fs*
L9 (00.59)
0.0 IES223-11ds
15.9 IES223-7ds
33.4 IES223-1ds**
46.4 IES223-8ds**
57.0 IES223-2ds**
L9 (00.60)
0.0 IES306-2fd
48.3 IES381-3fs
53.1 IES381-1fs
84.8 IES281-4fs**
L9 (00.61)
0.0 IES151-8ds**
13.2 IES151-9ds**
L9 (00.62)
0.0 IES16-4fd*
17.3 IES16-9fs**
39.2 IES16-8fs
50.9 IES16-2fs
L9 (00.63)
0.0 IES68-4fs**
22.3 IES68-5ds**
35.5 IES68-7fs
51.6 IES68-10fs**
L9 (00.64)
0.0 IES68-8ds**
4.8 IES68-11ds**
5.2 IES68-3fs**
18.0 IES176-5fs
31.9 IES32-4fs
62.3 IES68-6fs**
L9 (00.65)
0.0 IES323-6fs*
12.8 IES323-1fs
13.2 IES323-3fs
29.1 IES200-3ds
56.7 IES200-7ds**
58.1 IES200-8ds**
L9 (00.66)
0.0 IES394-4fs
14.3 IES394-2fs
21.4 IES394-1fs**
37.3 IES394-5ds**
53.3 IES29-10fs

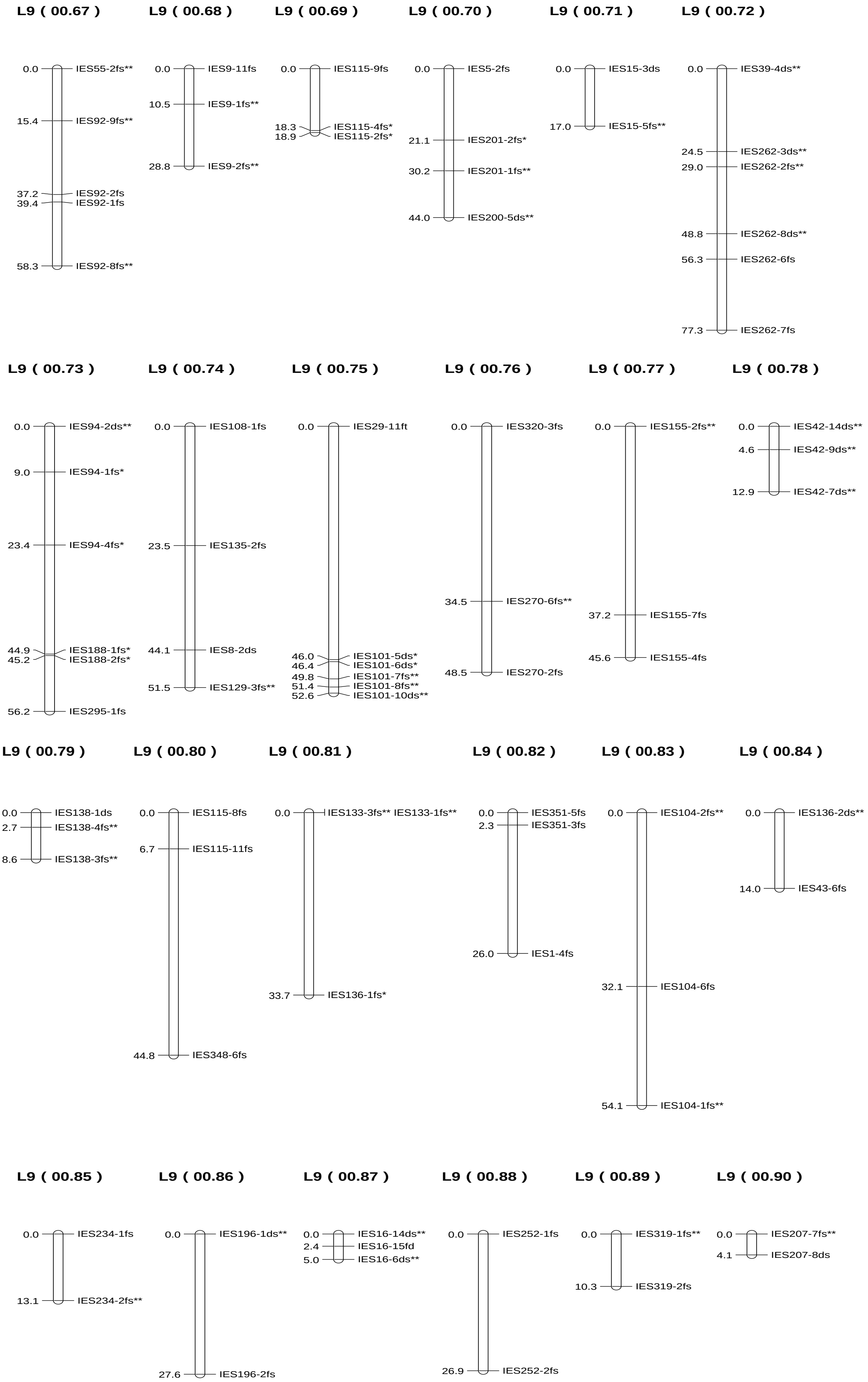
L9 (00.67)
0.0 IES55-2fs**
15.4 IES92-9fs**
37.2 IES92-2fs
39.4 IES92-1fs
58.3 IES92-8fs**
L9 (00.68)
0.0 IES9-11fs
10.5 IES9-1fs**
28.8 IES9-2fs**
L9 (00.69)
0.0 IES115-9fs
18.3 IES115-4fs*
18.9 IES115-2fs*
L9 (00.70)
0.0 IES5-2fs
21.1 IES201-2fs*
30.2 IES201-1fs**
44.0 IES200-5ds**
L9 (00.71)
0.0 IES15-3ds
17.0 IES15-5fs**
L9 (00.72)
0.0 IES39-4ds**
24.5 IES262-3ds**
29.0 IES262-2fs**
48.8 IES262-8ds**
56.3 IES262-6fs
77.3 IES262-7fs
L9 (00.73)
0.0 IES94-2ds**
9.0 IES94-1fs*
23.4 IES94-4fs*
44.9 IES188-1fs*
45.2 IES188-2fs*
56.2 IES295-1fs
L9 (00.74)
0.0 IES108-1fs
23.5 IES135-2fs
44.1 IES8-2ds
51.5 IES129-3fs**
L9 (00.75)
0.0 IES29-11ft
46.0 IES101-5ds*
46.4 IES101-6ds*
49.8 IES101-7fs**
51.4 IES101-8fs**
52.6 IES101-10ds**
L9 (00.76)
0.0 IES320-3fs
34.5 IES270-6fs**
48.5 IES270-2fs
L9 (00.77)
0.0 IES155-2fs**
37.2 IES155-7fs
45.6 IES155-4fs
L9 (00.78)
0.0 IES42-14ds**
4.6 IES42-9ds**
12.9 IES42-7ds**
L9 (00.79)
0.0 IES138-1ds
2.7 IES138-4fs**
8.6 IES138-3fs**
L9 (00.80)
0.0 IES115-8fs
6.7 IES115-11fs
44.8 IES348-6fs
L9 (00.81)
0.0 IES133-3fs** IES133-1fs**
33.7 IES136-1fs*
L9 (00.82)
0.0 IES351-5fs
2.3 IES351-3fs
26.0 IES1-4fs
L9 (00.83)
0.0 IES104-2fs**
32.1 IES104-6fs
54.1 IES104-1fs**
L9 (00.84)
0.0 IES136-2ds**
14.0 IES43-6fs
L9 (00.85)
0.0 IES234-1fs
13.1 IES234-2fs**
L9 (00.86)
0.0 IES196-1ds**
27.6 IES196-2fs
L9 (00.87)
0.0 IES16-14ds**
2.4 IES16-15fd
5.0 IES16-6ds**
L9 (00.88)
0.0 IES252-1fs
26.9 IES252-2fs
L9 (00.89)
0.0 IES319-1fs**
10.3 IES319-2fs
L9 (00.90)
0.0 IES207-7fs**
4.1 IES207-8ds
